# Nanocatalytic activity of clean-surfaced, faceted nanocrystalline gold enhances remyelination in animal models of multiple sclerosis

**DOI:** 10.1038/s41598-020-58709-w

**Published:** 2020-02-11

**Authors:** Andrew P. Robinson, Joanne Zhongyan Zhang, Haley E. Titus, Molly Karl, Mikhail Merzliakov, Adam R. Dorfman, Stephen Karlik, Michael G. Stewart, Richard K. Watt, Benjin D. Facer, Jon D. Facer, Noah D. Christian, Karen S. Ho, Michael T. Hotchkin, Mark G. Mortenson, Robert H. Miller, Stephen D. Miller

**Affiliations:** 1 Department of Microbiology-Immunology, Feinberg School of Medicine, Northwestern University, Chicago, IL USA; 2 Clene Nanomedicine, Inc., Salt Lake City, UT USA; 3 George Washington University, Washington, DC USA; 4 Lawson Health Research Institute, London Health Sciences Center, Ontario, Canada; 5 Brigham Young University, Provo, UT USA; 6 Vanderbilt University School of Medicine, Nashville, TN USA; 7 Sidney Kimmel Medical College at Thomas Jefferson University, Philadelphia, PA USA; 8 University of Utah School of Medicine, Salt Lake City, UT USA

**Keywords:** Pharmaceutics, Neurological disorders

## Abstract

Development of pharmacotherapies that promote remyelination is a high priority for multiple sclerosis (MS), due to their potential for neuroprotection and restoration of function through repair of demyelinated lesions. A novel preparation of clean-surfaced, faceted gold nanocrystals demonstrated robust remyelinating activity in response to demyelinating agents in both chronic cuprizone and acute lysolecithin rodent animal models. Furthermore, oral delivery of gold nanocrystals improved motor functions of cuprizone-treated mice in both open field and kinematic gait studies. Gold nanocrystal treatment of oligodendrocyte precursor cells in culture resulted in oligodendrocyte maturation and expression of myelin differentiation markers. Additional *in vitro* data demonstrated that these gold nanocrystals act via a novel energy metabolism pathway involving the enhancement of key indicators of aerobic glycolysis. In response to gold nanocrystals, co-cultured central nervous system cells exhibited elevated levels of the redox coenzyme nicotine adenine dinucleotide (NAD+), elevated total intracellular ATP levels, and elevated extracellular lactate levels, along with upregulation of myelin-synthesis related genes, collectively resulting in functional myelin generation. Based on these preclinical studies, clean-surfaced, faceted gold nanocrystals represent a novel remyelinating therapeutic for multiple sclerosis.

## Introduction

Myelination is a complex process by which axons are wrapped with oligodendrocyte (OL) membranes containing specialized proteins and lipids, facilitating axonal electrical conduction and providing essential trophic support to neurons^1^. During active myelination, OLs synthesize on the order of 100,000 proteins per minute^2^ and several thousand new lipid molecules per second^3^, reflecting the significant energetic investment needed for biomass generation, and making this cell type among the most energetically demanding of the body.

Dysregulated energy metabolism impacting OLs has been postulated to play a central role in MS disease progression^4,5^. Limited remyelination occurs in multiple sclerosis (MS) lesions despite the presence of OPCs in or around lesion sites. These OPCs fail to remyelinate and exhibit markers of metabolic stress^6^. Similarly, cultured human OLs, placed under metabolic stress using low glucose media, retract their cellular processes and exhibit significantly reduced glycolytic activity, favouring cell survival at the expense of myelin stability^7,8^. Under favourable growth conditions, human OPCs and OLs preferentially utilize aerobic glycolysis over oxidative phosphorylation for energy generation, resulting in an enhanced capacity to differentiate and myelinate axons^7^. Aerobic glycolysis results in production of pyruvate, acetyl coenzyme A, and NADPH, the requisite precursors of myelin proteins and lipids. The enhancement of the glycolytic pathway has been proposed as a potential therapeutic target for remyelination in MS^7^.

Here we describe a new nanocatalytic therapeutic candidate for remyelination consisting of a suspension of clean-surfaced, faceted nanocrystals of gold. Unlike bulk gold, which does not corrode and is generally recognized as chemically inert, gold at the nanoscale can be highly catalytic. Gold nanocatalysis is now widely used in many industrial applications^9^, but only recently have biologically-relevant catalytic properties of gold nanoparticles been described^10^. One of the key reactions catalysed by gold nanoparticles is the oxidation of nicotinamide adenine dinucleotide hydride (NADH) to the critical energetic co-factor, NAD^+^^11^. NAD^+^ and NADH are not only metabolic sensors of cellular energy levels, but also serve as the essential redox couple for ATP-generating reactions, oxidative phosphorylation and glycolysis^12^. In addition, NADH oxidation drives cellular respiratory and metabolic processes that play key roles in the energetically demanding process of myelination^7,8,13^.

A novel electro-crystallization-based method using pure gold wire, United States Pharmacopeia (USP) grade water, and sodium bicarbonate results in suspensions of gold nanocrystals of 13 nm average diameter, termed CNM-Au8. Because this method does not require the addition of organic capping or stabilization reactants, it results in clean, faceted nanocrystals. The method avoids the potentially toxic deposition of organic residues on nanoparticle surfaces made using traditional synthesis methods^14,15^. CNM-Au8 exhibited significantly higher nanocatalytic activity than that of other commercially available comparator gold particles.

The aim of our study was to determine whether stimulation and support of energy metabolic pathways in OLs by gold nanocatalysis results in remyelination of axons and recovery of behavioural functions in multiple preclinical models of MS. Here, we describe the efficacy of CNM-Au8 to promote the robust remyelination of demyelinated axons and restore motor function in *in vivo* animal models of MS, induce differentiation of OPCs, and enhance activities of neurons and OLs through enhancement of bioenergetic processes. Based on these results, CNM-Au8 is a viable therapeutic candidate for the treatment of remyelination failure in MS.

## Results

### Characterization of CNM-Au8

CNM-Au8 is a stable, aqueous suspension of gold (Au) nanoparticles in the shape of hexagonal bi-pyramids, pentagonal bi-pyramids, octahedrons, and tetrahedrons, as analysed by transmission electron microscopy (TEM). Each nanocrystal has approximately 68,000 Au atoms per nanocrystal with a 13 nm median diameter and a corresponding molar mass of ~1.3 × 10^4^ kDa. The faceted structure of a single gold nanocrystal produced by electrocrystallization is shown in Fig. S1 and nanocrystal characteristics are summarized in Table S1.

**Figure 1:**
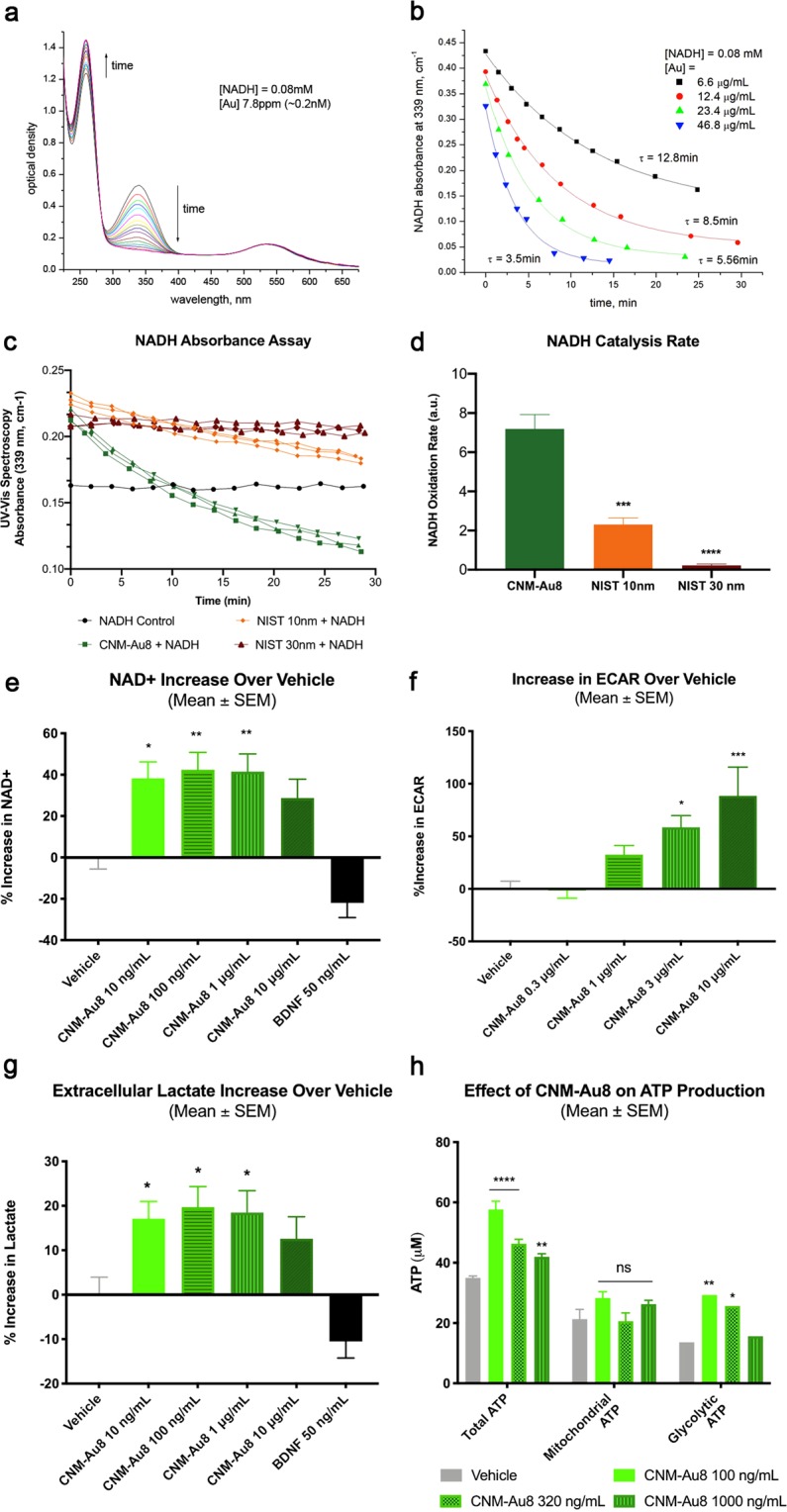
Nanocatalysis by CNM-Au8 enhances cellular bioenergetics. (**a**) Changes in absorbance peaks of NADH (339 nm) and NAD^+^ (259 nm) demonstrate the conversion of NADH to NAD^+^ with time in the presence of 6.6 μg/mL CNM-Au8. Shown is an overlay of UV-Vis spectra taken at approximately 1 min intervals; total elapsed time of more than 60 minutes. Starting concentration of NADH at t = 0 was 0.08 mM. (**b**) Dose-dependent catalytic activity of CNM-Au8 (6.6 μg/mL black squares, 12.4 μg/mL red circles, 23.4 μg/mL green triangles, 46.8 μg/mL blue triangles) on the oxidation of NADH as measured by change in absorbance of NADH over time. (**c**) Catalytic activity of CNM-Au8 compared to two NIST standards, NIST 10 nm (orange) and NIST 30 nm (red), using the same starting concentrations: 3.4 μg/mL Au added to 26 μM NADH in 5.7 mM NaHCO_3_. No gold control (black) shows stability of 26 μM NADH during the same time frame. (**d**) Initial rates of catalysis for CNM-Au8, NIST 10 nm, and NIST 30 nm, calculated from curves shown in B. (**e**) Effect of CNM-Au8 treatment on NAD^+^ levels, expressed as percent change over vehicle, in primary mesencephalic cultures compared to vehicle (grey), or BDNF control. (**f**) Extracellular acidification rate (ECAR) of purified murine OLs in response to CNM-Au8 in the first three minutes following glucose challenge as measured in the Seahorse flux analyser, expressed as percent change over vehicle. (**g**) Extracellular lactate levels in media of primary rat mesencephalic cultures after 48 h treatment with CNM-Au8, expressed as percent change over vehicle. (**h**) Total, mitochondrial, and glycolytic intracellular ATP levels from human OL M03.13 cells treated with vehicle (grey) or CNM-Au8 (green). (**d**–**g**) One-way ANOVA, corrected for multiple comparisons. (**h**)Two-way ANOVA. Quantities shown are group means +/− SEM. *p < 0.05; **p < 0.01; ***p < 0.001; ****p < 0.0001.

Colloidal gold nanoparticles have previously been shown to catalyze the oxidation of NADH to NAD^+^ in a cell-free system^11^. Utilizing this UV-Vis spectrometry assay^11^, we demonstrated that CNM-Au8 efficiently catalyzed the oxidation of NADH to NAD^+^ (Fig. 1a–d). Figure 1a shows the time course of the change in absorbance peak of NADH at 339 nm with a concomitant increase in the NAD^+^ peak at 259 nm. The control, sampled over the same time period without the addition of CNM-Au8, showed no spectral changes in NADH absorbance, demonstrating that NADH alone was stable and did not oxidize under these conditions (Fig. 1c, black curve). Furthermore, the oxidation of NADH by CNM-Au8 was dose-dependent; Fig. 1b shows NADH absorbance at 339 nm as a function of time for four concentrations of CNM-Au8 (6.6, 12.4, 23.4, and 46.8 µg/mL). We compared the catalytic activity of CNM-Au8 to that of two reference standards of gold nanoparticles from the U.S. National Institute of Standards and Technology (NIST), 10 nm and 30 nm in diameter, respectively, at equivalent concentrations. Under the same reaction conditions, the nanocatalytic activity of CNM-Au8 significantly exceeded the catalytic activity of the citrate-reduced gold nanoparticle standards of both diameters by over 3-fold (Fig. 1c,d).

Similarly, CNM-Au8 treatment of living cells increased the intracellular NAD^+^ pool. Primary co-cultures of neural and glial cells from the mesencephalon of day 15 rat embryos were exposed to CNM-Au8, brain derived neurotrophic growth factor (BDNF, a control for neuroprotection), or vehicle, for 36 hours. Figure 1e demonstrates that total cellular NAD^+^ levels were elevated following treatment with CNM-Au8.

The increase in NAD^+^ suggested that glycolytic and anabolic processes may be stimulated by CNM-Au8 treatment. To measure elevated glycolytic activity, an extracellular acidification rate (ECAR) assay was used to demonstrate the effect of CNM-Au8 treatment in OLs immediately following glucose challenge (Fig. 1f). Figure 1g shows that CNM-Au8 treatment of primary neuronal-glial co-cultures resulted in elevated levels of extracellular lactate, which is produced as a result of elevated glycolytic activity. Further, an increase in non-mitochondrial ATP production would be expected to result from enhanced glycolytic activity. As shown in Fig. 1h, ATP derived from glycolytic activity significantly contributed to the enhanced levels of total ATP in CNM-Au8-treated human OLs compared to the vehicle controls.

### Remyelination by nanocrystalline gold using the *in vivo* cuprizone model of demyelination

The copper-chelating toxin cuprizone selectively induces the apoptosis of mature OLs in the central nervous system (CNS) of mice following a standard treatment protocol^16^. With five weeks of cuprizone feeding, maximal demyelination of axons in the corpus callosum can be achieved, serving as a model for MS as well as other white matter degenerative diseases^16^. To determine whether CNM-Au8 had a therapeutic effect on mice exposed to cuprizone, CNM-Au8 was delivered to randomized groups of mice (N = 15 per group) by gavage (10 mg/kg/day) (Groups 1–5) or was provided as their drinking water *ad libitum* (Groups 6 and 7). A prophylactic treatment arm consisted of providing CNM-Au8 to Groups 4 and 6 concomitant with the start of CPZ treatment, while a therapeutic treatment arm consisted of starting CNM-Au8 dosing two weeks after cuprizone administration had been initiated (Groups 5 and 7). Group 1 served as a non-cuprizone-treated vehicle control (sham), with vehicle provided by gavage daily. To assess the time-course of cuprizone damage, Group 2 was started on cuprizone and vehicle by gavage, then sacrificed after two weeks to verify the ongoing cuprizone insult. Group 3 was a cuprizone and vehicle control that was sacrificed at the end of five weeks when maximal myelin damage was expected to have taken place^16^. A schematic for the study design is shown in Fig. S2.

**Figure 2:**
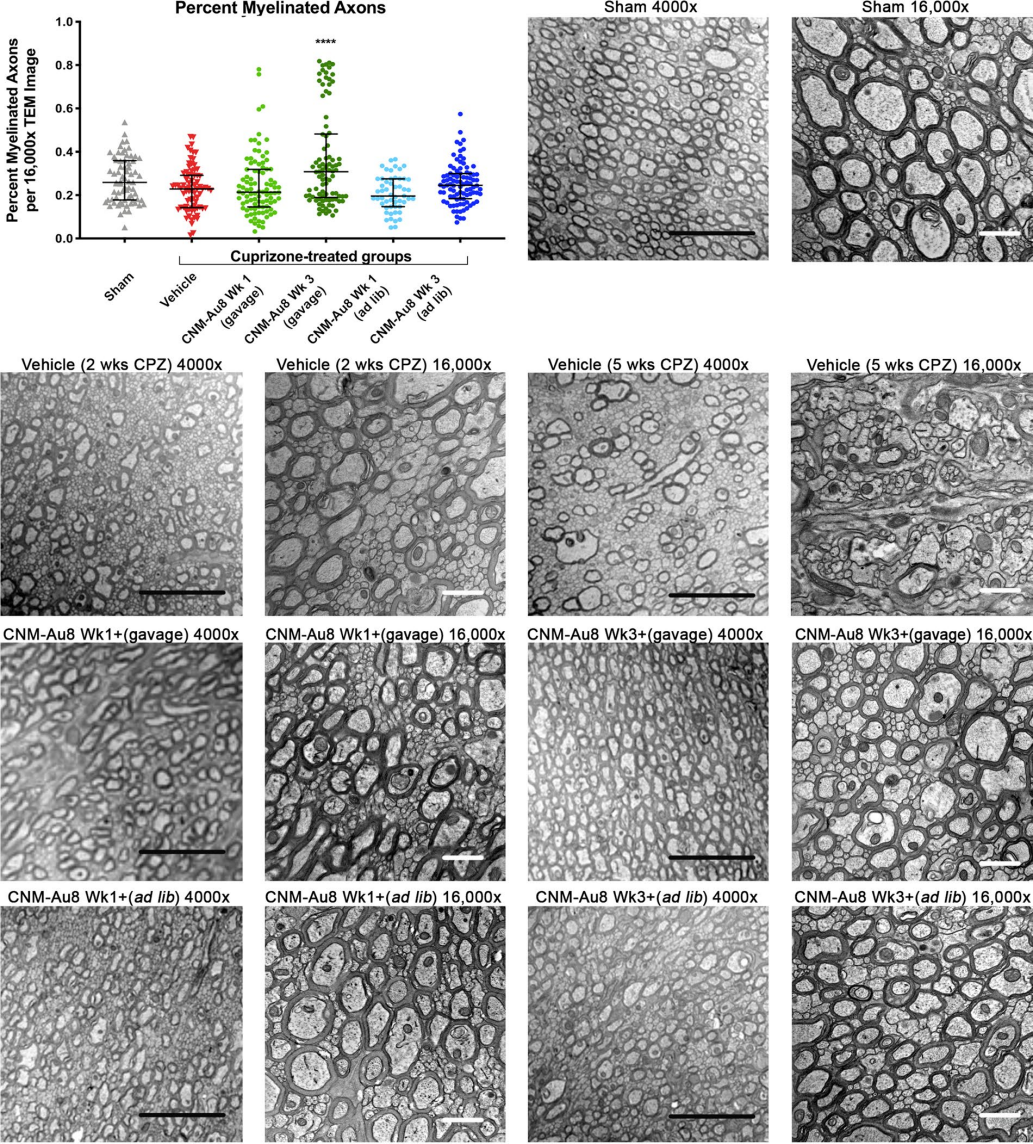
Effect of CNM-Au8 in an *in vivo* cuprizone model of demyelination. Scatterplot (upper left) shows a quantitation of the percent myelinated axons using 16,000x TEM images by treatment group. Six of the seven experimental groups are shown; animals treated with cuprizone and vehicle for only two weeks were not quantitated. Bars show mean and SEM of each group. ****p < 0.0001, one-way ANOVA corrected for multiple comparisons. Representative TEM images of corpus callosum axons in cross section from sham-treated Group 1 animals, cuprizone-treated animals sacrificed at Week 2 (“Vehicle (2 wks CPZ)”), cuprizone-treated animals sacrificed at Week 5 (“Vehicle (5 wks CPZ)”), cuprizone plus gavage-dosed CNM-Au8 treated animals from the prophylactic arm starting at week 1 (“Wk 1+”), cuprizone plus gavage-dosed CNM-Au8 treated animals from the therapeutic arm starting at week 3 (“Wk 3+”), cuprizone plus ad libitum-dosed CNM-Au8 treated animals from the prophylactic arm, and cuprizone plus ad libitum-dosed CNM-Au8 treated animals from the therapeutic arm. Magnifications (4000x and 16,000x) are as labelled; black scale bar = 5 μm; white scale bar = 1 μm.

To quantitate remyelination activity following CNM-Au8 treatment, the number of myelinated and unmyelinated axons in each image was counted using ImageJ (N = 587 images; average of 84 images per treatment group). As expected, cuprizone treatment reduced the percentage of myelinated axons compared to sham treated animals (Fig. 2, scatterplot p = 0.0221, two-tailed Mann-Whitney test). Striking recovery of myelinated axons was observed for therapeutically treated animals who were dosed with CNM-Au8 by gavage (Fig. 2, dark green circles). An increase in myelinated axons was also observed for animals in the prophylactic arm with CNM-Au8 administered by gavage; however, this trend did not reach statistical significance when corrections for multiple comparisons were applied (Fig. 2, light green circles). Animals dosed with CNM-Au8 *ad libitum* also displayed improved myelination in TEM images (Fig. 2); however, this did not reach statistical significance.

TEM images of the corpus callosum of animals from each group were also analyzed qualitatively in a blinded fashion (Fig. 2). After two weeks of cuprizone treatment, significant changes in myelin morphology were noted. In comparison to the ordered array of well-packed and ovoid-shaped, myelinated axons in the vehicle-treated control (Fig. 2, “Sham”), cuprizone feeding caused extensive CNS damage: the tissue appeared disorganized, frequent delamination of lamellae was apparent, axons were irregularly-shaped, and patches of demyelinated axons were observed within areas of normal-appearing myelination (Fig. 2, “Vehicle (2 wks CPZ)” and “Vehicle (5 wks CPZ)”). By contrast, in cuprizone-fed animals treated with CNM-Au8, there were extensive areas of normal myelin observed, as well as very few swollen myelin sheaths and a reduced incidence of delamination (Fig. 2).

These results corroborated those of an earlier pilot cuprizone experiment in which four groups of mice, randomized by body weight (N = 4 per group), were treated with vehicle, cuprizone, CNM-Au8 alone, or CNM-Au8 and cuprizone (Fig. S3). In this study, CNM-Au8 treated animals exhibited higher numbers of myelin-wrapped axons (Fig. S3b) in the corpus callosum compared to vehicle controls, and G-ratio analysis of these images revealed a distinct population of newly myelinated axons, (Fig. S3c). CNM-Au8 treated animals also exhibited higher levels of myelin proteolipid protein (PLP) expression (Fig. S3d). Taken together, these demyelinating animal model studies indicated that CNM-Au8 treatment consistently resulted in higher levels of myelinated axons compared to vehicle treated controls.

### Remyelination by CNM-Au8 does not occur via inactivation of cuprizone

To rule out the possibility that CNM-Au8’s activities are cuprizone-dependent, for example by blocking cuprizone’s copper-binding activity, UV-Vis spectroscopy was used to monitor binding activities of copper to cuprizone in the presence and absence of CNM-Au8. Copper chelation by cuprizone was characterized by an absorbance peak at 600 nm (Fig. S4a,b). Addition of CNM-Au8 to cuprizone alone resulted in a slight redshift in the absorbance curve (Fig. S4c), suggesting a weak surface interaction of CNM-Au8 with cuprizone. However, the absorbance peak of the copper-cuprizone saturation curve in the presence of CNM-Au8 remained unaltered at 600 nm, demonstrating uninhibited interaction of cuprizone with copper in the presence of CNM-Au8 (Fig. S4d,e). When the background signal due to CNM-Au8 alone was subtracted, the absorption spectrum of copper-cuprizone complexes coincided with that of copper-cuprizone without CNM-Au8, demonstrating that the presence of CNM-Au8 did not affect the sequestration of copper by cuprizone (Fig. S4d,e).

**Figure 3:**
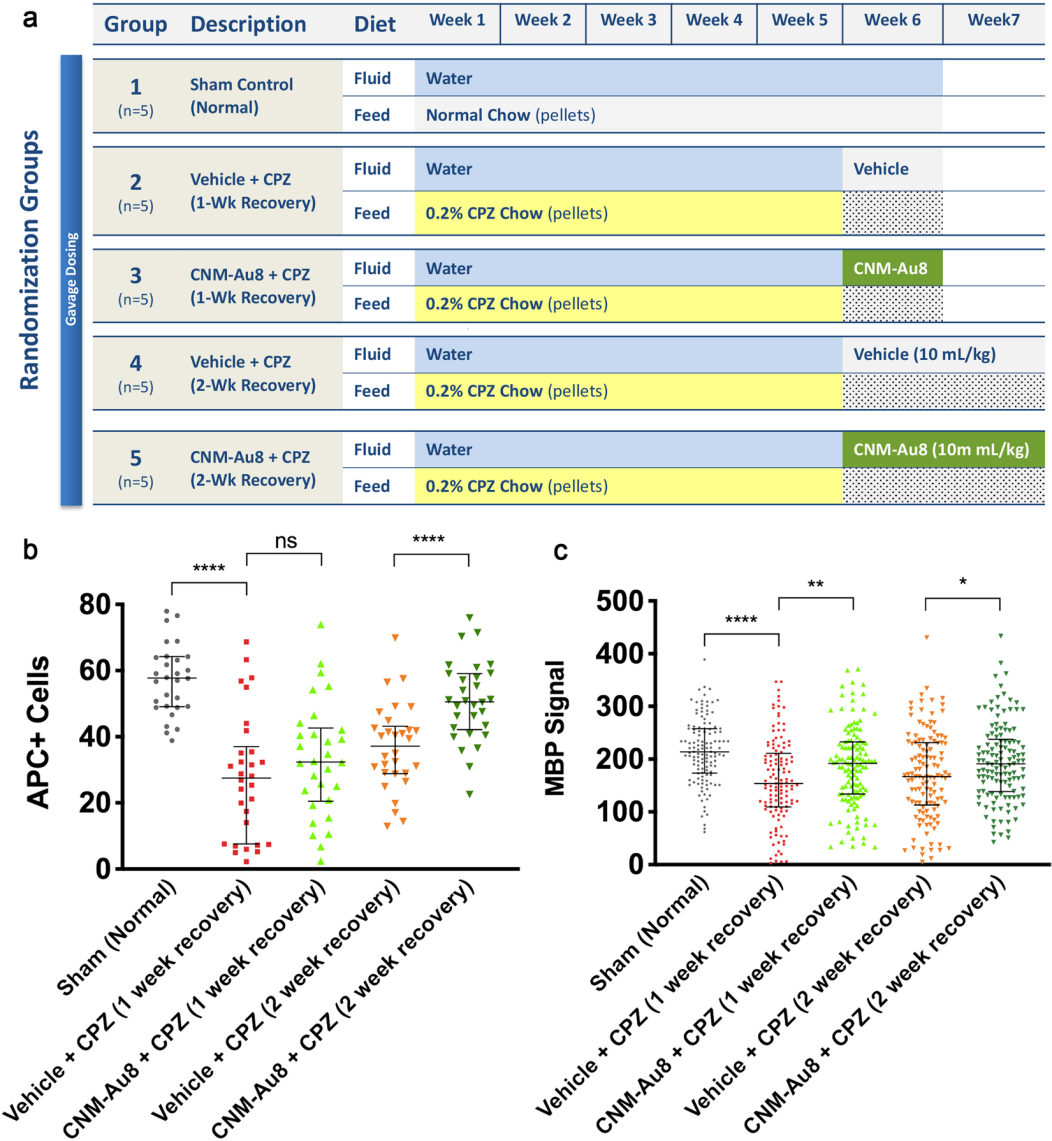
CNM-Au8 promotes oligodendrocyte maturation when administered post-cuprizone treatment. (**a**) Experimental design schematic. (**b**) Quantitation of the number of APC positive cells by immunohistochemical staining of corpus callosum coronal sections from each treatment group. (**c**) Quantitation of the area of anti-MBP immunohistochemical staining of corpus callosum coronal sections from each treatment group. (**b**,**c**), Error bars show median and interquartile range.

To assess the *in vivo* efficacy of CNM-Au8 once cuprizone was withdrawn, mice (N = 5 per group) were fed cuprizone for five weeks, then cuprizone was discontinued, and treatment with CNM-Au8 or vehicle commenced and continued for the following week or two weeks (Fig. 3a). Figure 3b,c show that CNM-Au8 stimulated myelin marker expression in OLs after cuprizone was withdrawn. The number of mature, myelin-producing OLs was assessed by antibody staining coronal sections from each mouse using anti-APC (CC1), a marker of mature OL cell bodies, and with anti-MBP, a marker of myelin-producing OLs, at the end of either one or two weeks of treatment with CNM-Au8 compared with vehicle controls. As shown in Fig. 3b, the animals treated with CNM-Au8 for two weeks following cuprizone insult showed an increase in the number of APC-positive cells in comparison with vehicle-treated control, which reached statistical significance for the two-week, CNM-Au8-treated group. This is supported by MBP immunohistochemistry results shown in Fig. 3c, in which an increase in MBP staining in CNM-Au8-treated mice over their respective vehicle controls is observed.

### Remyelination by CNM-Au8 does not occur by direct protection of mature OLs from cuprizone-mediated apoptosis

Cuprizone preferentially induces the apoptosis of mature OLs over OPCs and other neural cell types^16^. To determine whether CNM-Au8 acts to protect mature OLs from apoptosis, mice were simultaneously treated with cuprizone and CNM-Au8 or vehicle control for two weeks during the initial onset of cuprizone OL toxicity, after which the animals were sacrificed and coronal brain sections were stained for mature OLs using anti-APC and anti-MBP antibodies (Fig. S5). Early assessment of the effects of cuprizone during this two-week period allowed us to detect a potential reduction in mature OL numbers before OPCs could differentiate and replenish the mature OL population.

After two weeks of cuprizone treatment, the number of OLs marked by anti-APC and MBP was reduced in all coronal sections, and this reduction was not improved by concurrent treatment of CNM-Au8 or prophylactic treatment of CNM-Au8 starting one week prior to cuprizone treatment (Fig. S5). This observed reduction in mature OL numbers and MBP expression indicated that CNM-Au8 does not protect mature OLs from cuprizone-induced toxicity. Recovery of myelin following CNM-Au8 treatment in cuprizone-treated animals is therefore not due to the improved survival of mature OLs, but more likely due to enhanced differentiation of OPCs.

### Effects on motor functions in cuprizone-treated mice in open-field and movement kinematic behavioral assays

**Figure 4:**
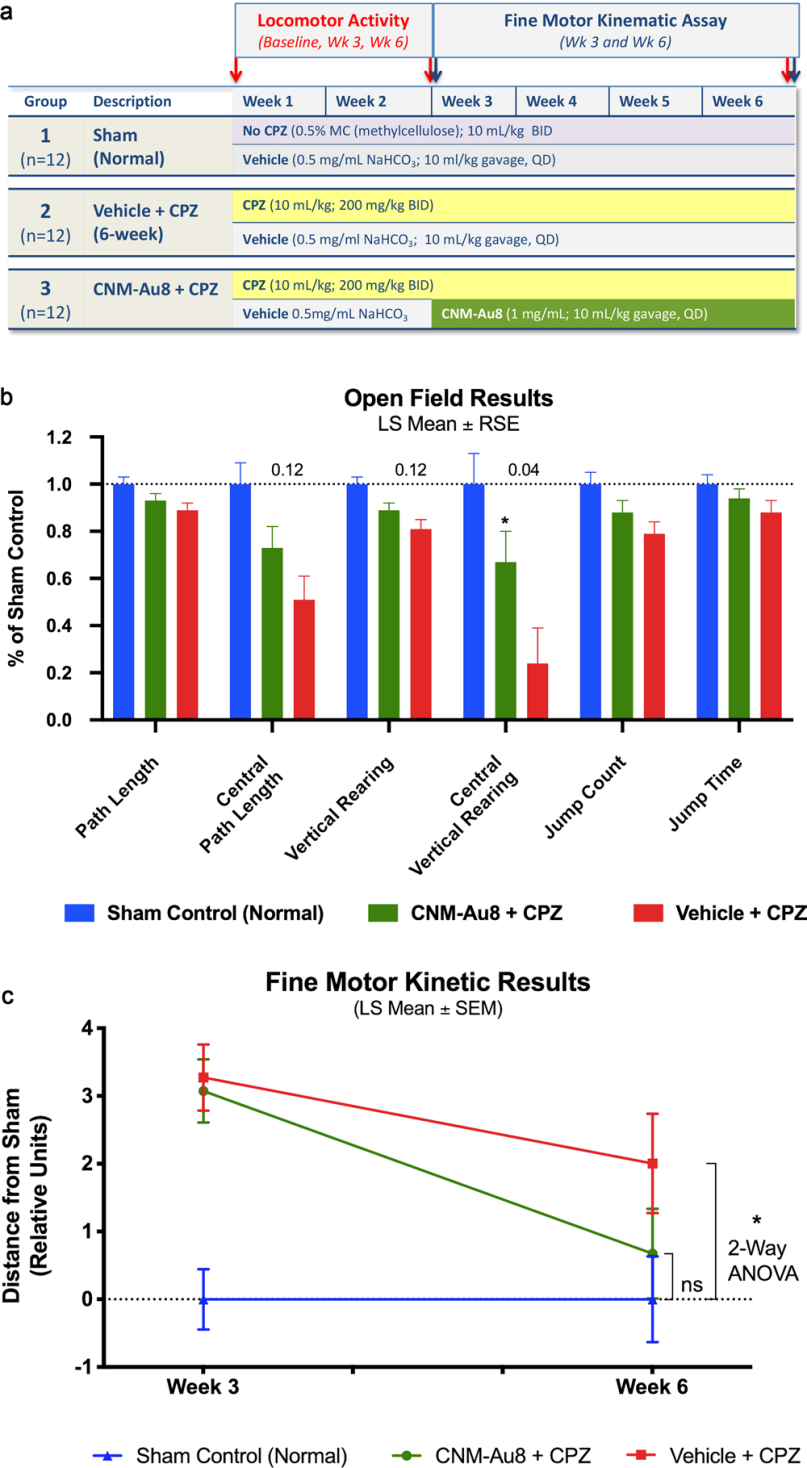
Restoration of function by CNM-Au8 in open field test and fine motor kinematic analyses. (**a**) Schematic of this study design. (**b**) Quantitation of parameter measures from open field assessments of behaviour in untreated sham mice (blue bars), gavage-administered CNM-Au8 treated animals with cuprizone (green bars), and gavage-administered vehicle treated animals with cuprizone (red bars) expressed as a percentage of sham measurements at Week 6. (**c**) Principal component analysis of gait metrics showed no statistical difference (p = 0.47) between CNM-Au8 and sham treated groups as compared to a detectable difference in vehicle treated groups vs. sham (p = 0.032; 2-way ANOVA) by week 6. See Materials and Methods for statistical calculations.

To determine whether CNM-Au8 treatment was capable of restoring function after cuprizone insult, open field gross motor assessments and fine motor kinematic assessments were conducted on groups of mice (N = 12 per group, open field; N = 10 per group, fine motor kinetics) treated with CNM-Au8 or vehicle. Group 1 was a non-cuprizone control with vehicle delivered to animals by gavage starting on Day 1. Group 2 animals were administered cuprizone and vehicle by gavage for six weeks; Group 3 was administered cuprizone by gavage for six weeks and CNM-Au8 for the final four weeks of the six-week study period. Behaviors for the open field test were assessed prior to cuprizone dosing, at the end of week 3, and at the end of week 6, while the fine motor kinematic tests were assessed at the end of week 3, and at the end of week 6 (Fig. 4a).

In eight of the nine quantitative behavioral parameters that were assessed in the open field test, cuprizone-treated animals showed statistically significant behavioral deficits from sham-treated animals by the end of six weeks. The ninth metric, average speed, was unchanged between cuprizone-treated and the sham non-cuprizone treated controls, and was therefore not evaluated further. Figure 4b shows the change in each behavioral parameter for each group at week 6 compared to baseline as a least-square mean result. Overall, CNM-Au8 treatment of cuprizone-injured animals improved the performance of the mice in each of the open field test assays, restoring the observed cuprizone deficits versus sham treated animals, even though most individual behavioral metrics did not reach statistical significance. More specifically, we observed between 43% to 62% relative recovery toward sham behavior in each parameter due to CNM-Au8 treatment versus vehicle cuprizone treated animals (Table S2), and in all cases there was a trend in improvement towards sham. There was a statistically significant improvement in central vertical rearing counts (p < 0.05), and near-significant trends in central path length and vertical rearing counts (Fig. 4b).

In addition to the open field test, fine motor kinematic analyses were conducted in which parameters measuring limb joint movements, posture, and gait were measured using high-speed cameras that filmed each mouse from multiple viewpoints (top, bottom, and side) as the animal moved. One hundred individual movement parameters were measured from these videos for a quantitative assessment of gait at 3 weeks and 6 weeks after the start of cuprizone treatment. Daily dosing of CNM-Au8 for the treated group was started at Week 3. Principal component analysis of these parameters, plotted as distance from sham at Weeks 3 and Week 6, is shown in Fig. 4c. At Week 6, the cuprizone- treated animals displayed significant gait impairment in comparison to the untreated sham group (−2.0 ± 0.9 units, p = 0.032 vs. sham, 2-way ANOVA). In contrast, treatment with CNM-Au8 largely restored kinematic movements to normal levels as the CNM-Au8-treated group became statistically indistinguishable from sham-treated controls at 6 weeks (−0.67 ± 0.9 units; p = 0.468 vs. sham). Between week 3 and week 6, CNM-Au8-treated mice improved by 78% from their week 3 deficit relative to the sham controls.

### Increased OL migration to lesion sites and increased myelin production by nanocrystalline gold following lysolecithin induced demyelination

**Figure 5:**
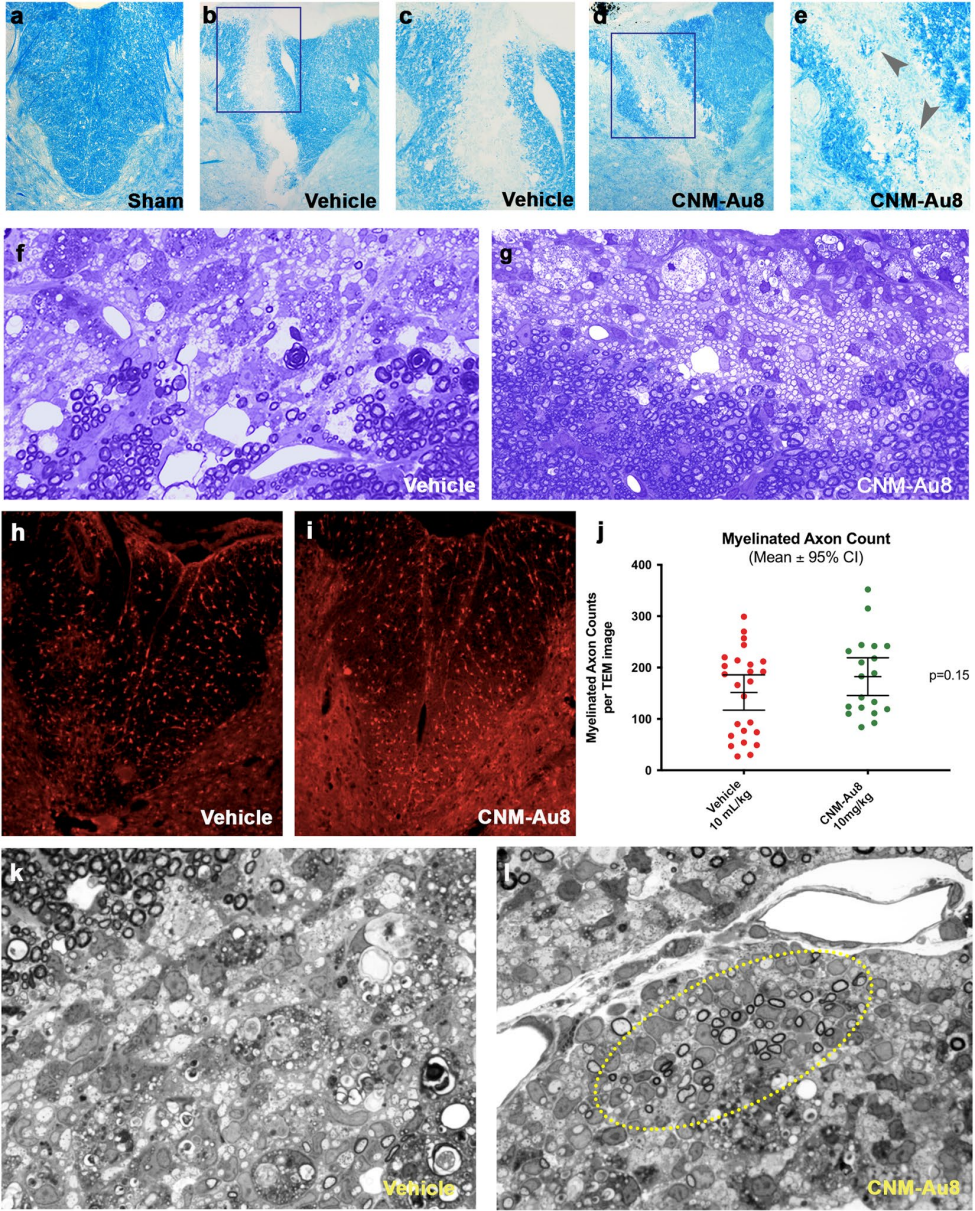
Evidence of remyelination at seven and fourteen days post-lesion by CNM-Au8 treatment in a focal demyelination lysolecithin rodent model. Following lysolecithin lesion on Day 0, animals were sacrificed for histological and electron microscope analyses on Day 7 and Day 14, following daily gavage dosing of CNM-Au8 (10 mg/kg/da) or vehicle. (**a**–**e**) Luxol Fast Blue staining of myelin in spinal cord sections of lesions at Day 7: from sham treated animals (**a**); lysolecithin lesioned, vehicle treated animals (**b**,**c**); and lysolecithin lesioned, CNM-Au8 treated animals (**d**,**e**), in which recovery of myelin within lesion area of CNM-Au8 treated animals (**d**,**e**) can be seen (grey arrowheads). (**a**,**b**,**d**) 10x magnification; (**c**,**e**): 20x magnification. (**f**,**g**) toluidine blue staining of myelin in spinal cord sections of lesions at Day 14 from vehicle (**f**) and CNM-Au8 (**g**) treated animals showing the distinct ‘honeycomb’ pattern of remyelination in CNM-Au8-treated animals; (**f**,**g**): 63x magnification. (**h**,**i**) anti-APC staining of mature OL cell bodies within spinal cord sections of lesions from vehicle (**h**) and CNM-Au8 (**i**) treated animals. Clusters of mature OLs can be observed in the lesion area of CNM-Au8 treated animals (**i**). (**j**) Quantitation of myelinated axon counts from TEM images of spinal sections of lesions by animal showed a 43% increase in myelination in treated animals over vehicle controls (p = 0.15, unpaired t-test). (**k**,**l**), Representative high resolution 100x images of spinal cord lysolecithin lesions of animals treated with vehicle (**k**) and with CNM-Au8. (**l**) Normal myelination, at the boundary of the lesion, can be observed in each panel at the upper margins of the field of view. Evidence of thinly-wrapped remyelinating axons are shown in (**l**) (encircled by yellow dashed line).

Stereotactic lysolecithin injection is an acute, focal method used to induce demyelination of the spinal cord. To determine the effect of CNM-Au8 on remyelination in this model, the lysolecithin detergent lysophosphatidylcholine (LPC) was injected into the dorsal white matter of thoracic (T7) spinal cords of rats (N = 15 per group). Group 1 was therapeutically treated with CNM-Au8 by gavage three days post-LPC injection and Group 2 was similarly administered vehicle control. Animals were sacrificed for histological or immunohistochemical staining on Day 14 following LPC injection. Histological sections of rat spinal cords at the site of injection were stained with either Luxol Fast Blue (LFB) at day-7 (Fig. 5a–e) or toluidine blue at day-14 (Fig. 5f,g) to visualize myelin. LFB staining revealed the presence of new myelin within the site of the lesion in sections of the CNM-Au8-treated animals (Fig. 5d,e) as compared to the vehicle-treated controls (Fig. 5b,c). Similarly, toluidine blue staining of spinal cord sections from CNM-Au8-treated animals (Fig. 5f,g) revealed a striking ‘honeycomb’ pattern of newly myelinated axons within the lesion site, in comparison with a lack of evidence of new myelin in vehicle-treated controls (Fig. 5g). APC (CC1) staining of lysolecithin-induced spinal cord lesions revealed clusters of viable, mature OLs within lesion sites in CNM-Au8-treated animals which were again lacking in vehicle treated controls (Fig. 5h,i).

Thinly myelinated axons, which are those with 2–4 wraps of myelin located within lesion sites, indicate axons undergoing remyelination post-LPC injection. A quantitation of thinly myelinated axons within lesion sites was conducted in a blinded fashion using TEM image analyses. The results of the quantitation are shown (Fig. 5j) and indicated that CNM-Au8-treated animals exhibit a 43% mean increase in myelinated axons within lesions post-LPC injection compared to vehicle controls. Representative TEM images clearly show evidence of remyelinating axons within lesion sites of CNM-Au8 treated animals compared to vehicle controls (Fig. 5k,l).

### *In vitro* differentiation of OPCs with treatment of nanocrystalline gold

**Figure 6:**
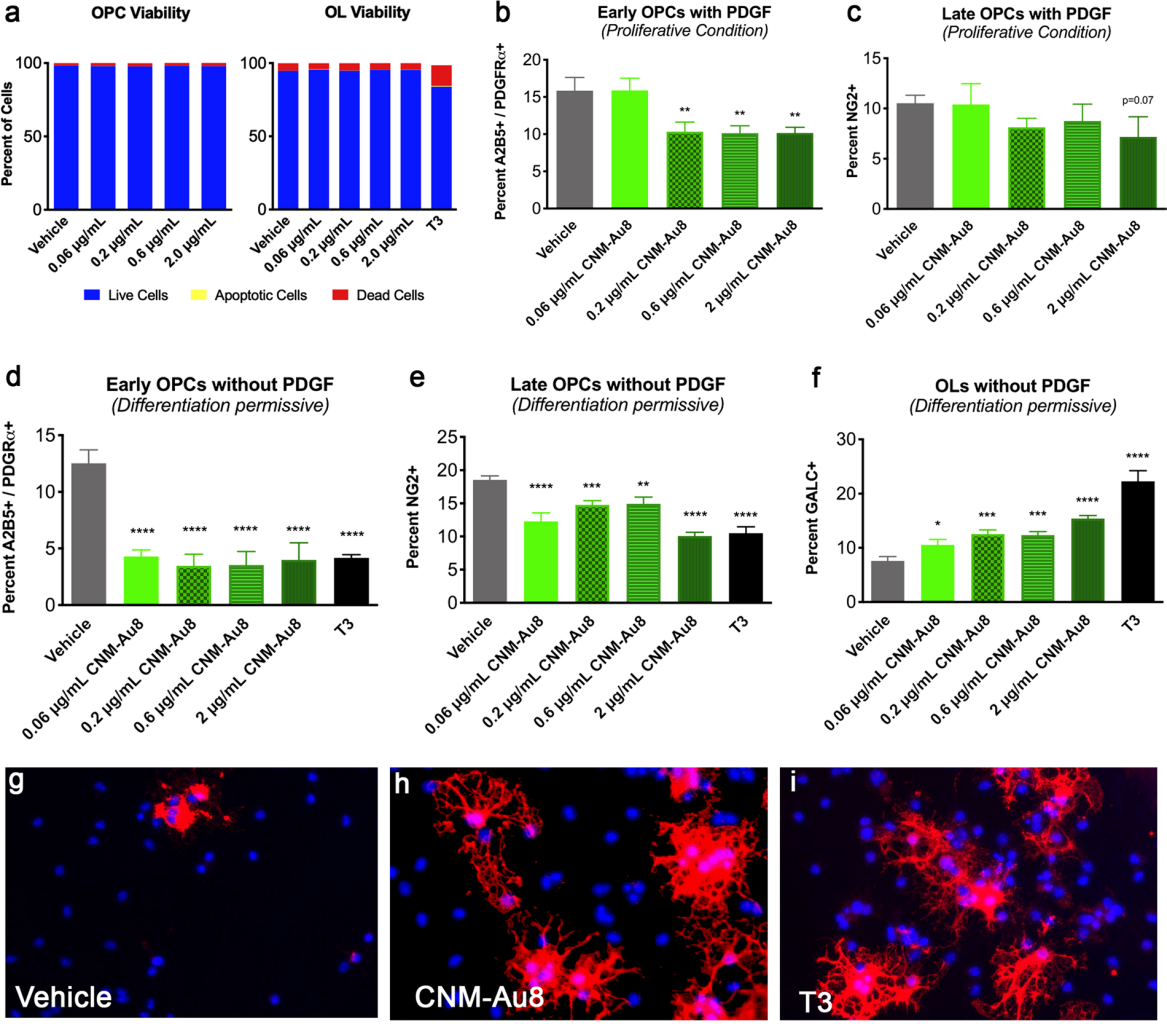
CNM-Au8 mediated differentiation of immunopanned primary OPCs in culture. (**a**) Increasing concentrations of CNM-Au8 did not affect OPC or OL viability in culture. (**b**,**c**) OPCs supplemented with 0.01 μg/mL PDGF, ‘proliferative conditions,’ do not show a proliferative response when provided with CNM-Au8 in the media compared to vehicle treated controls; cells expressing markers for early (**b**) and late (**c**) OPCs show a relative decline in numbers in response to increasing concentrations of CNM-Au8. (**d**–**f**) OPCs grown without PDGF, under ‘differentiation-permissive conditions,’ show a relative increase in cell numbers expressing the mature OL marker GALC (**f**), while showing a relative decrease in numbers of OPCs expressing early (**d**) and late (**e**) OPC markers compared to vehicle treated controls. (**g**–**i**) representative images of OPCs treated with vehicle (**g**), 0.6 μg/mL CNM-Au8 (**h**) and 0.04 μg/mL T3 (**i**) stained with DAPI to mark cell nuclei and anti-MBP to mark OLs expressing the mature myelin marker. OPCs treated with CNM-Au8 and cells treated with the positive control T3 showed higher numbers of mature OLs with complex cytoplasmic networks indicative of differentiated OLs.

The cuprizone and lysolecithin experiments suggested that CNM-Au8 either enhanced the proliferation of OPCs, or enhanced the differentiation of OPCs into OLs, or both. In order to address these possibilities, we utilized a flow cytometry OL differentiation assay^17^ in which OPCs were isolated^18^ and cultured in the presence of CNM-Au8 or vehicle. First, viability of OPCs in the presence of CNM-Au8 or vehicle was assessed. OPCs and differentiated OLs were grown in the presence of 0.06, 0.2, 0.6, and 2 µg/mL CNM-Au8 for four days and cell viability was measured using annexin V and propidium iodide stains to differentiate live, apoptotic, and dead cells. No change in viability of OPCs or OLs in response to CNM-Au8 treatment was observed (Fig. 6a).

Flow cytometry results indicated that CNM-Au8, similar to the known OPC differentiation signaling molecule triiodothyronine (T3), enhanced differentiation of mature OLs at the expense of early OPCs (Fig. 6b,c). OPCs were treated for four days with CNM-Au8 in growth media with (Fig. 6b,c) or without (Fig. 6d–f) the proliferation inducer PDGF, stained with antibodies to mark early OPCs (A2B5+/PDGFRα+) or late OPCs (NG2+) and then separated based on marker expression and quantitated by flow cytometry. CNM-Au8 elicited a clear differentiation response from OPCs when cultured in medium without PDGF (Fig. 6d–f). Flow cytometry based on markers for early OPCs (A2B5+/PDGFRα+), late OPCs (NG2+) and mature OLs (GALC+) showed a pronounced shift toward mature OLs in response to CNM-Au8 treatment, as indicated by proportionately higher populations of GALC + OLs in CNM-Au8-treated cultures (Fig. 6f). Immunohistochemical staining of OLs for MBP further confirmed not only the presence of more MBP+ OLs in response to CNM-Au8, but also that the terminally differentiated cells take on the morphological characteristics of differentiated OLs in the form of extensive networks of processes to a similar extent and degree as the differentiation signaling molecule, T3 (Fig. 6g–i).

A second study further confirmed that CNM-Au8 stimulated the differentiation of OLs regardless of whether they were derived from mouse CNS or rat spinal cord cultures. Rat spinal cord cells were dissociated, cultured, and treated with CNM-Au8 or vehicle for 72 hours and then stained with DAPI, as well as antibodies against Olig2 to mark OLs and MBP to mark cell bodies and processes of mature OLs (Fig. S6).

### Differential gene expression analysis of CNM-Au8-treated OPC cultures *in vitro*

**Figure 7:**
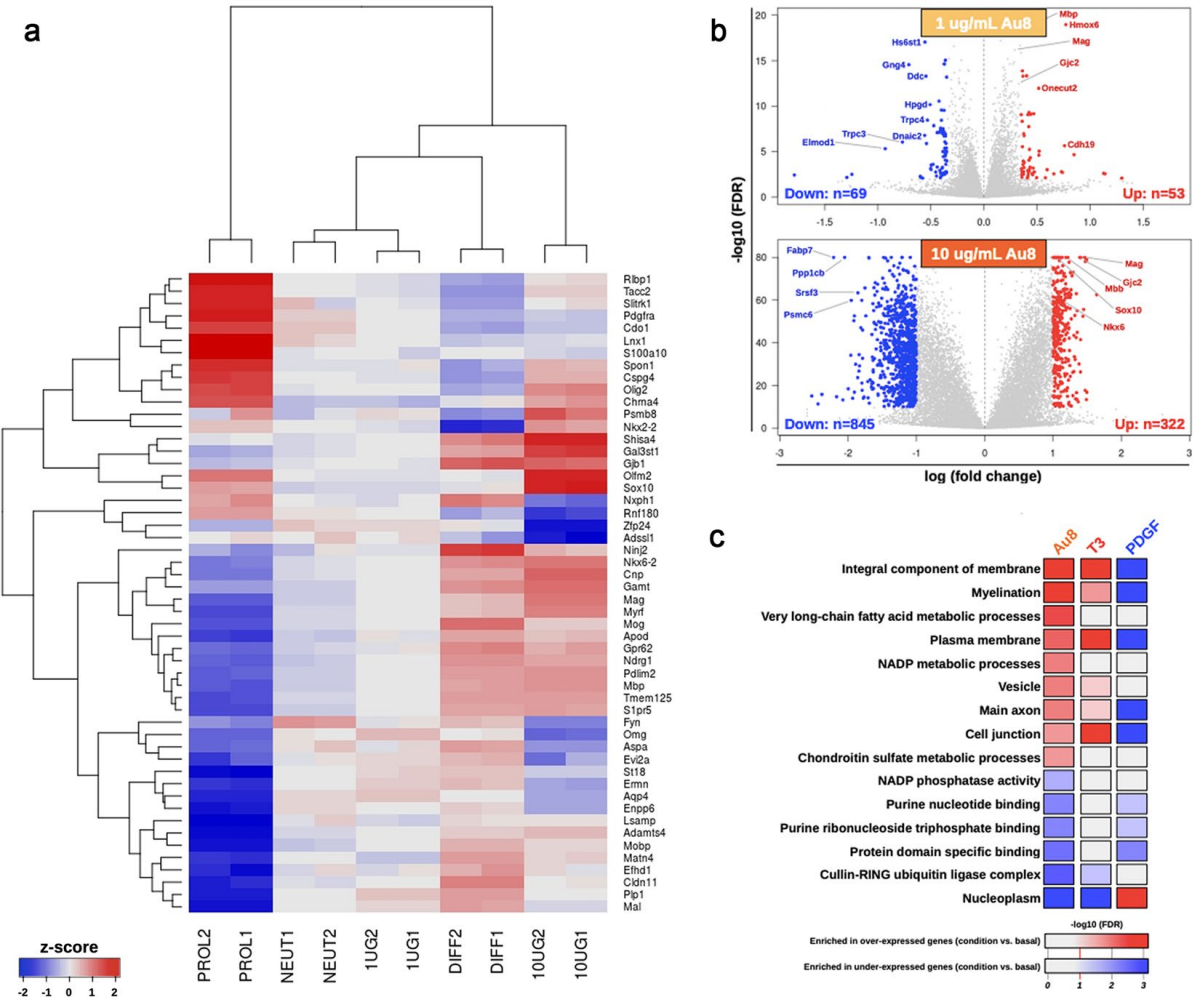
Transcriptomics analyses identified OL differentiation and myelination pathways that are upregulated in response to CNM-Au8. Isolated OPC cultures were treated with vehicle (NEUT1 and NEUT2), 1.0 (1UG1 and 1UG2) or 10.0 μg/mL (10UG1 and 10UG2) CNM-Au8, 0.01 μg/mL PDGF to induce proliferation (PROL1 and PROL2), or 0.04 μg/mL T3 (DIFF1 and DIFF2) to induce differentiation, for 72 h in duplicate. Cells were then processed for RNAseq analyses. (**a**) The top fifty variable genes in the dataset are displayed in the heatmap, with the name of each gene in each row labeled to the right. Samples were hierarchically clustered (dendrogram at top), demonstrating that CNM-Au8 treated cells’ transcript profiles are closer, but not overlapping, that of T3-treated cells, as compared to PDGF treated cells. (**b**) Volcano plots of differentially expressed genes, with the identities of upregulated genes known to play a role in differentiation and myelination indicated in red. (**c**), Gene ontology enrichment analysis revealed that gene pathways associated with myelination, plasma membrane regulation, and fatty acid metabolism were among those upregulated upon treatment with CNM-Au8, as well as, in part, with T3.

To further characterize the effect of CNM-Au8 on OPC differentiation, we conducted an *in vitro* RNAseq expression study using isolated, purified OPC cultures, with each treatment condition performed in triplicate (Fig. 7). Multidimensional scaling analysis was used to examine relative distances between samples according to their gene expression profiles. These analyses demonstrated that 1 µg/mL and 10 µg/mL CNM-Au8-treated OPC expression profiles were more similar to the expression profile of differentiated OLs treated with T3, than to the profile of proliferating OPCs treated with PDGF (Fig. 7a). Furthermore, CNM-Au8 treatment of mouse OPCs in primary culture for 72 hours results in differential expression (DE) of genes involved in myelination. Markers of OL maturation such as *Mag*, *Mbp*, *Gjc2*, *Nkx6*.*2*, and *Sox10* mRNAs were elevated at least 2-fold over untreated vehicle controls (Fig. 7b). Notably, there was also an enrichment of mRNA transcripts with gene ontologies related to lipid metabolism uniquely present in the DE gene profile for CNM-Au8-treated OPCs (Fig. 7c). These included genes encoding proteins involved in long chain fatty acid synthesis, which is essential to the generation of lipids that comprise ~70% of myelin. In contrast, the DE gene profiles for the T3 and PDGF controls did not demonstrate enrichment in long chain fatty acid synthesis mRNAs. A Table of DE genes supporting the pathways in Fig. 7c is provided in Table S3. Taken together, these data demonstrate that treatment with CNM-Au8 promotes OPC differentiation and OL maturation.

Our initial bioinformatics analyses of the glycolytic, ATP-generating, and common energy metabolic pathways indicated that key genes known to be regulators of glycolysis/TCA cycle were not upregulated in the RNAseq experiment with the exception of *Keap1* (*Kelch-like ECH-associated protein 1*), which exhibited a positive fold change of 1.709-fold in the CNM-Au8-treated samples over vehicle-treated samples. Transcripts corresponding to the Krebbs cycle rate-limiting enzyme Isocitrate dehydrogenase (*Idh1*), as well as known metabolic regulators of energy metabolism and glycolysis: *Nrf2* (*Nfe2I2*), *Prkaa*1 (*Ampk*), *Pfkfb2*, *Pdha1*, and *Pdhb*, were down-regulated, with fold changes ranging between 0.486 to 0.914-fold. Significantly, transcripts encoding the glycolytic regulator, *Pfkfb2* (phosphofructo-2-kinase/fructose-2,6-bisphosphatase 2)^19^ showed substantial repression (0.613-fold). We then conducted an undirected bioinformatics approach utilizing all significant results for upregulated transcripts (1.2-fold or higher) using Reactome Pathway Browser^20^. Using this approach, we found that a highly significant overrepresented upregulated pathway (p = 6.50E-04) was the *Hsf1* (*heat shock factor 1*)-dependent transactivation pathway, with the following twenty upregulated transcripts supporting this pathway enrichment, including: *Akt1s1*, *Rptor*, *Tnfrsf21*, *Serpinh1*, *Hspa1b*, *Hspa1a*, *Col4a6*, *Fkbp4*, *Cryab*, *Mtor*, *Camk2b*, *Hsf1*, *Ubb*, *Mlst8*, *Hspb8*, *Hspb1*, *Hspb2*, *Slc29a1*, *Hspa2*, *Dedd2*. Previous studies have demonstrated that Hsf1 function resides at the nexus of NAD+ -associated metabolic energy regulation, cellular stress responses, and regulators of myelination (e.g, mTor)^21^. Results of this RNASeq study therefore supported the hypothesis that CNM-Au8 treatment induces an energetic response in OPCs, leading to the expression of myelination transcripts, albeit not directly through the upregulation of canonical genes associated with glycolysis, but rather through the activation of the heat shock factor pathway that coordinates energy metabolism, myelination^22^, and cellular responses to stress^21^.

## Discussion

More than a decade of materials science and nanotechnology research has revealed unexpected catalytic properties of gold at the nanoscale. In addition to oxidation of NADH to NAD^+^ first characterized by Huang *et al*.^11^ and discussed above, gold nanoparticles have demonstrated peroxidase, oxidase, catalase, and super oxide dismutase-like properties, all of which have significant relevance to biologic systems^23,24^. We now propose that a novel preparation of clean-surfaced faceted crystalline gold has direct therapeutic ability to support intracellular bioenergetic activities.

A novel electrocrystallization process that, importantly, does not involve any capping or stabilizing agents, was devised to grow highly faceted, pure gold crystals. This new method afforded several significant advantages: (1) the nanocatalytic activity of CNM-Au8 was significantly enhanced compared to that of standard citrate-reduced gold nanoparticles (Fig. 1c), (2) CNM-Au8 nanocrystals were non-toxic to OPC and OL cells in culture (Fig. 6a), and (3) CNM-Au8 functionally improved motor behaviors in mice *in vivo* following exposure to the demyelinating agent, cuprizone (Fig. 4). In multiple *in vivo* remyelination assays, treatment with CNM-Au8 significantly improved not only the quantifiable detection of myelinated axons in the brains of experimental animals, but also mouse behaviors and functional movements in the open field test and kinematic assays. In contrast, the necessary addition of capping and surfactant agents in order to generate and stabilize nanoparticles made using traditional methods can introduce adverse, cytotoxic effects in biological systems^15^.

The currently available FDA-approved drugs for the treatment of MS act to suppress the recurrent autoimmune attack on myelin during disease progression. These drugs generally limit disease progression by reducing the frequency and intensity of autoimmune attacks. A remyelinating therapy, acting in concert with, or independently of, immunosuppressant drugs, opens up the possibility of restoration of functions that were previously impaired or lost due to MS disease activity, thereby improving patients’ quality of life and potentially reversing disease progression.

CNM-Au8 appears to act through a novel nanocatalytic mechanism through a pathway involving the energetic coenzymes NAD^+^ and NADH. These metabolites are vital for driving cellular reduction-oxidation (redox) reactions in living cells, including both glycolytic and oxidative phosphorylation pathways of energy generation. In addition to its redox role, NAD^+^ acts as a substrate for key regulators of metabolism and repair^25^. NADPH is generated from NAD^+^, and is required for anabolic pathways including the synthesis of long chain fatty acids that are required not only for myelin production, but also to protect cells from reactive oxygen species (ROS)^26^.

A NAD^+^ deficiency of more than 50% has been detected in the serum samples of MS patients compared to healthy controls^27^. Intriguingly, this NAD^+^ deficiency increases amongst MS subgroups with increasing severity of the disease^27^. NAD^+^ has been proposed to be a potential therapeutic target for MS treatment^27–29^.

OPCs present in MS lesions are thought to be unable to remyelinate because they have undergone cellular stresses that have led to their bioenergetic failure (3). CNM-Au8 may catalytically compensate for intracellular energy loss, stimulating OPC differentiation and accelerating myelination^28,29^.

Many neurodegenerative diseases share hallmarks of energy depletion, mitochondrial dysfunction, and metabolic stress in multiple central nervous system cell types prior to clinical symptom onset^30^. Because the mechanism of action of CNM-Au8 is not limited to a single protein target or cell type in the CNS, the applicability CNM-Au8 as a neurotherapeutic is potentially wide-ranging and highly applicable to those neurodegenerative diseases impacted by impaired cellular bioenergetics. Preclinical studies investigating the therapeutic potential of CNM-Au8 for the treatment of Parkinson’s disease, amyotrophic lateral sclerosis, and other complex neurodegenerative diseases are presently underway.

There are limitations to this study. While we demonstrated an improvement in open field and fine motor behaviors in response to CNM-Au8 in cuprizone-treated mice, many of the individual parameters measured did not reach statistical significance distinct from the cuprizone-treated controls (Fig. 4). Post-hoc sample size calculations based on the observed differences between the Group 1 (sham) animals and the Group 2 (vehicle plus cuprizone-treated) animals indicated that significantly more animals would be needed to detect significant treatment effects on individual parameters. Cuprizone itself does not consistently demyelinate brain areas uniformly when evaluated both on a within-animal and a between-animal basis^31^; therefore the toxin’s effects, particularly if mild, can be difficult to demonstrate on a quantitative basis. Nevertheless, remyelination due to CNM-Au8 treatment was reproducibly demonstrated in the four cuprizone and the lysolecithin models presented herein, when evaluated on ultrastructural, cellular, and functional levels. The observed functional and gait improvements with oral administration of CNM-Au8 therefore warrants further study.

In addition to the preclinical efficacy studies of CNM-Au8 as a potential remyelinating therapeutic for MS described here, the drug development program for CNM-Au8 included IND-enabling toxicology studies as well as a First-in-Human Phase I study (NCT02755870). The results of the genotoxicity, safety pharmacology, and acute and chronic toxicology studies for six months’ duration in rodents and nine months’ duration in canines indicated a clean safety profile for CNM-Au8, with no severe adverse effects identified in any of these studies even at the highest maximum feasible doses tested.

Notably, the blood brain barrier penetrance of CNM-Au8 was demonstrated as part of these chronic toxicity studies by determining the concentration of CNM-Au8 in the spinal cord and brain of canines dosed with CNM-Au8 by oral gavage for nine months. Quantitation of CNM-Au8 in organs and tissues is confounded by the presence of naturally-occurring background levels of gold. Nevertheless, quantitation by inductively coupled plasma mass spectroscopy demonstrated significantly more animals with CNS gold levels greater than the limit of detection in the spinal cord, CSF, and brains when dosed with CNM-Au8 by gavage daily compared to vehicle controls. Therefore, orally delivered CNM-Au8 is blood brain barrier penetrant, albeit at low levels.

Due to promising preclinical data described here and the favorable safety/toxicity profiles achieved by CNM-Au8, a Phase 2, double-blinded, randomized, placebo-controlled study, VISIONARY-MS (Treatment of Visual Pathway Deficits In Chronic Optic Neuropathy to Assess the Efficacy, Safety, Tolerability, and Pharmacokinetics of CNM-Au8 For Remyelination In Stable Relapsing Multiple Sclerosis (RMS) is currently underway. This clinical study is designed to assess remyelination through a functional vision improvement primary endpoint, change in low contrast letter acuity, and a secondary electrophysiologic endpoint of improvement in multi-focal visual evoked potential latency in the affected eye. These endpoints have been employed in previous clinical trials for the assessment of remyelinating activity^32–34^. Results from VISIONARY-MS are expected in 2021.

## Methods

### Animal care and husbandry

Animal studies were approved by the respective Institutional Animal Care and Use Committees at Charles River Labs (Kuopio, Finland) and Northwestern University Feinberg School of Medicine, and conducted with adherence to the NIH Guide for the Care and Use of Laboratory Animals. Animals were housed under pathogen–free conditions with food and water provided *ad libitum*. C57BL/6 mice and Sprague-Dawley rats were used in all experiments.

### Vehicle and CNM-Au8

The vehicle used was 6.5 mM NaHCO_3_.

The processes for production of CNM-Au8 have been described (US Patent 9,603,870)^35^. In brief, water containing NaHCO_3_ at a 6.5 mM concentration was transferred to an electrochemical processing device through which the buffered water flowed at a constant rate. The buffered water was exposed to an electrical plasma generated between a gold electrode, suspended above the water, and the surface of the buffered water, which surface functioned as a second electrode to generate “conditioned water.” The conditioned water was subsequently exposed to a series of paired gold wire electrodes. Each gold wire electrode pair was held by a controller that advanced the electrode pairs through the conditioned water with continuous application of alternating current as the conditioned water flowed past each electrode pair. The resulting gold nanocrystal suspension was subsequently concentrated, filtered with an antimicrobial filter, then filled into single unit HDPE containers. All processes were executed in a clean room. Each batch of CNM-Au8 was analytically assayed (Table S1) to ensure release specification standards were met.

### Cell-free NADH oxidation assays

UV-Vis spectroscopy was conducted as described^11^. Citrate-reduced 30 nm and 10 nm gold nanoparticles were acquired from the National Institute of Standards and Technology.

### *In vitro* quantitation of NAD+ and NADH

Primary rat mesencephalic neural-glia co-cultures were seeded at a density of 40,000 cells/well in 96-well plates pre-coated with poly-L-lysine^36^. After four days of culture, CNM-Au8 (10 ng/mL, 100 ng/mL, 1 µg/mL, 10 µg/mL), BDNF (50 ng/mL), or vehicle was added for 36 h. Quantitation of NAD^+^ and NADH was performed by bioluminescent assay (Promega kit #G9071).

### *In vitro* quantitation of ATP

M03.13 cells (TebuBio) were seeded at a density of 20,000 cells/well in a 96-well plate. After one day of culture, cells were treated with CNM-Au8 (0.1 µg/mL, 0.32 µg/mL, 1 µg/mL) or vehicle for 72 h. Half of the wells (N = 3 per condition) were treated with mitochondrial blockers (100 µM antimycin A, 0.5 µM rotenone, 3 µM oligomycin) for two hours. Wells were then lysed in luciferase buffer. ATP was determined by bioluminescence assay (1 s integration time, CLARIOstar). Mitochondrial ATP was calculated by subtracting the average ATP level from mitochondria-blocked cells from average total ATP levels of untreated cells.

### Extracellular acidification rate (ECAR) measurements

OPCs from P7 mouse pups were immunopanned and seeded in 96-well plates in OPC optimal medium^18^. CNM-Au8 (0.3 ng/mL, 1 ng/mL, 3 ng/mL, 10 ng/mL) or vehicle was added to the culture for 24 h. ECAR was measured using the Seahorse flux analyser (Agilent). Protein content was determined by Bradford (BioRad). Experiments were performed in triplicate.

### Cuprizone treatment

The 5-week cuprizone demyelination model has been described^16^. Male mice (Taconic Farms, 8 weeks old) were assigned to one of seven groups, balanced by weight, N = 15 animals in each group. 0.2% cuprizone chow and normal control chow were obtained from Harlan Laboratories (Bethesda, MD). Groups 1–5 were dosed with vehicle (6.5 mM NaHCO_3_ at 10 mL/kg by gavage daily) or CNM-Au8 (10 mg/kg/day) by gavage at the same time (+/− 1 hour) each day. Cuprizone treatment started on Day 0 for all groups except control Group 1, which was fed normal chow. Prophylactic dosing was started on Day 0 (Groups 1–4 and Group 6). Therapeutic dosing started on Day 14 (Groups 5 and 7). Group 2 mice were sacrificed on Day 14; all other groups were sacrificed on Days 34–36.

For the post-cuprizone CNM-Au8 treatment study, twenty-five 8-week old, male mice were divided by weight into five groups. For treatment schematic for each group, see Fig. 2a.

### Corpus callosum sectioning and TEM imaging

Two 2 mm sagittal slices of brain tissue on either side of the midline containing the posterior corpus callosum were dissected and processed for TEM imaging^37^ from mice from each treatment group. Imaging was carried out at 4000x, 16,000x, and 40,000x using Zeiss Libra 120 transmission electron microscope.

### Coronal brain sectioning, fixing, immunohistochemical staining and quantitation of signal

10 µm coronal mouse brain sections containing the corpus callosum (10 per animal) from each treatment group were prepared for immunohistochemical staining as described^38^. Sections were blocked with donkey serum and stained with 5 µg/ml MBP (Abcam, ab40390) or 1 µg/ml APC (Abcam, ab15270) overnight at 4 °C, washed in PBS, then stained with AlexaFluor 488 or AlexaFluor 594 (Millipore, 1:500), and counterstained with Hoechst. Imaging was done on a Nikon A1R+ confocal microscope. Post-image analysis of 8 sections per animal was conducted using ImageJ^39^.

### Qualitative assessment of TEM images

Analyses of 3000 TEM images (4000x, 13,500x, 16,000x, and 40,000x) from 85 mice (10–15 animals per treatment group) were carried out by a pathologist blinded to the treatment group.

### Quantitation of myelination

TEMs of corpus callosum images at 16,000x magnification: N = 587 images total; 70 images from Group 1 (N = 7 animals), 110 from Group 2 (N = 15 animals), 79 from Group 3 (N = 8 animals), 94 from Group 4 (N = 9 animals), 90 from Group 5 (N = 9 animals), 53 from Group 6 (N = 7 animals), and 91 from Group 7 (N = 7 animals) were analysed using ImageJ^39^ to count the number of myelinated and unmyelinated axons in each image. The percentage of myelinated axons per image was then calculated. G-ratios were calculated as the inner myelin circumference divided by the outer myelin circumference. Analyses were conducted by investigators blinded to treatment group.

### Lysolecithin demyelination model

The demyelinating lysolecithin lesion model has been described^40^. Animals were allowed to recover for 7 or 14 days prior to analysis. Vehicle (N = 15 rats) or CNM-Au8 (N = 15) was administered by gavage daily (10 mg/kg/day) starting on day 3 following lysolecithin injection. Animals were sacrificed on day 7 or day 14. Lesioned areas were sectioned and stained for myelin using Luxol fast blue or toluidine blue. Quantitation of myelinated axon counts was conducted on TEM images of lesion sections by manually counting the number of thinly-wrapped (2–4 wraps) axons within lesion boundaries, in blinded fashion.

### Open field and fine motor kinematic studies

Open field (N = 12 mice/group) and fine motor kinematic (N = 10 mice/group) analyses of cuprizone/CNM-Au8 treated animals were performed by Charles River Laboratories (Kuopio, Finland).

For the open field test, mice were placed in the centre of an activity chamber equipped with infrared beams (Med Associates, Inc.). Behaviours were recorded for 30 min in 5-min bins.

Fine motor skills and gait parameters were assessed using an automated, high precision kinematic analysis system (MotoRater, TSE Systems). Before each test session, mice were externally marked on joints of limbs and tail. Movements were captured using high speed (300 fps) cameras positioned above, below, and at the side of the animal and analysed using SimiMotion and custom software (Charles River Labs).

### Proliferation/differentiation assays of OPCs by flow cytometry

OPCs from stage P5-7 C57BL/6 mouse pups were immunopanned^18^, then grown in media supplemented with 0.01 μg/mL PDGF or 0.04 μg/mL T3 and treated with vehicle or CNM-Au8 for 72 hours. Flow cytometry was performed as described^17^.

### RNASeq transcriptomic expression assay

OPCs from stage P10 C57BL/6 mouse pups were immunopanned^18^, then treated with vehicle, 0.04 μg/mL T3, 0.01 μg/mL PDGF, or 1 μg/mL or 10 μg/mL CNM-Au8 for 72 hours. Two technical replicates were conducted for each condition.

RNAseq libraries were prepared from treated OPCs, then sequenced (Illumina Hiseq. 4000). Reads were aligned using STAR; gene counts were computed by *htseq-count*. Downstream analyses were performed using R, with the *limma*, *edgeR*, *TopGO* Bioconductor packages. Heatmaps were plotted using the *gplots* R package. Bioinformatics analyses were also conducted using Reactome Pathway Browser^20^. Pathways that were over-represented using the set of DE genes that were significantly upregulated (1.2-fold or higher) or downregulated (0.8-fold or lower) were analysed for relevance to energy metabolism, redox regulation, and myelination.

### Statistical methods

Unless described below, statistical analyses were carried out using MS Excel package and/or Prism 7.0. Statistical significance was defined as p values of 0.05 or less.

#### Open field parameters analysis

Three time points (Baseline, Week 3, Week 6) associated with each of six parameters (Path Length, Central Path Length, Vertical Rearing, Central Vertical Rearing, Jump Counts, and Jump Time) were analysed. Mixed model repeated measures (MMRM) was used to test for treatment differences (change from baseline). Covariates consisted of baseline score of each parameter and baseline weight. An unstructured covariance model was used to account for correlated measures within a subject. The Kenward-Roger method was used to compute degrees of freedom for the test of fixed effects. Least-square (LS) means and 95% confidence interval (CI) for each treatment and time point were calculated to account for the unbalanced data of the main effects and covariates. Comparisons of treatment groups were taken as the difference of the LS means.

#### Mouse motor kinematics

Ten principal components (PC) of gait features (Slowness, Tail Tip Height, Knee Function, Step Length, Interlimb Coordination, Vertical Movement, Hip Angle, Hindlimb Trajectory, Hip Height, Forelimb Trajectory) were identified from ~100 kinematic gait parameters collected. Each PC encompassed the results for 2 timepoints, Week 3 and Week 6.

To test for treatment differences, MMRM analyses were used in the same manner described above. Comparisons of treatment groups were taken as the difference of the LS means.

## Supplementary information

Supplementary information.

## Data Availability

CNM-Au8 must be obtained through an MTA. All data associated with this study are available in the main text or the Supplementary Materials.
