# Supplementary material for: Nanocatalytic activity of clean-surfaced, faceted nanocrystalline gold enhances remyelination in animal models of multiple sclerosis
Source: Sci Rep. 2020 Feb 11;10:1936. doi: 10.1038/s41598-020-58709-w (PMC7010780; doi:10.1038/s41598-020-58709-w)
Supplement: Supplementary file 1 — Supplementary information. [file 41598_2020_58709_MOESM1_ESM.pdf]

Supplementary Information

Nanocatalytic activity of clean-surfaced, faceted nanocrystalline gold enhances remyelination in animal models of multiple sclerosis

Authors:

Andrew P. Robinson[1]†, Joanne Zhongyan Zhang[2]†, Haley E. Titus[1], Molly Karl[3], Mikhail Merzliakov[2], Adam R. Dorfman[2], Stephen Karlik[4], Michael G. Stewart[5], Richard K. Watt[5], Benjin D. Facer[6], Jon D. Facer[5], Noah D. Christian[7], Karen S. Ho[2,8*], Michael T. Hotchkin[2]‡, Mark G. Mortenson[2]‡, Robert H. Miller[3]‡, Stephen D. Miller[1]‡

†These authors contributed equally to this work.

‡These authors contributed equally to this work.

Affiliations:

[1]Department of Microbiology-Immunology, Feinberg School of Medicine, Northwestern University, Chicago, IL.

[2]Clene Nanomedicine, Inc., Salt Lake City, UT.

[3]George Washington University, Washington DC.

[4]Lawson Health Research Institute, London Health Sciences Center, Ontario, Canada.

[5]Brigham Young University, Provo, UT.

[6]Vanderbilt University School of Medicine, Nashville, TN.

[7]Sidney Kimmel Medical College at Thomas Jefferson University, Philadelphia, PA.

[8]University of Utah, Salt Lake City, UT.

***To whom correspondence should be addressed:**

Karen S. Ho, karen@clene.com, 3165 Millrock Drive, Suite 325, Salt Lake City, UT 84121.

Supplementary Information

Fig. S1. TEM image of a representative 13 nm pentagonal bipyramidal gold nanocrystal

Fig. S2. Experimental design schematic to demonstrate effect of CNM-Au8 in an *in vivo* cuprizone model of demyelination

Fig. S3. Evidence of remyelination by CNM-Au8 in a pilot *in vivo* cuprizone mouse model

Fig. S4. CNM-Au8 did not inhibit cuprizone from chelating copper

Fig. S5. Loss of Apc+ OLs was unaffected by CNM-Au8 treatment within the first two weeks of cuprizone treatment

Fig. S6. Differentiation of OLs from primary rodent spinal cord cultures by CNM-Au8

Table S1. CNM-Au8 nanocrystal characterization

Table S2. Functional improvements in each open field parameter toward Sham controls by CNM-Au8 treated groups compared to vehicle controls.

Table S3. RNASeq DE genes identified overrepresenting the pathways shown in Fig. 7c

Supplementary Methods

Cuprizone experiments

Cuprizone experiments described in the Supplemental section were performed as described in the main paper Materials and Methods, with the following changes:

For the pilot cuprizone experiment, sixteen mice were divided into four groups with N=4 per group. Group 1 served as a negative control with vehicle provided *ad libitum* in drinking water and normal chow. Group 2 was fed 0.2% cuprizone chow with vehicle provided *ad libitum* in drinking water. Group 3 received CNM-Au8 (50 µg/mL) in their drinking water, and normal

chow. Group 4 received CNM-Au8 (50µg/mL) in their drinking water, and 0.2% cuprizone chow. After five weeks of treatment, animals were prepared for TEM imaging as described. The average intake of CNM-Au8 in the treatment groups was measured on a daily, per cage basis. From this data, a daily dose of 10 mg CNM-Au8/kg/day was calculated to be a sufficient dose for detectable effects on myelin.

For the early sacrifice cuprizone experiment, four groups of mice were treated as follows: Group 1 (N=4) was given normal chow and vehicle (10mL/kg) by gavage; Group 2 (N=6) was given 0.2% cuprizone chow and vehicle by gavage; Group 3 (N=6) was given 0.2% cuprizone chow and CNM-Au8 by gavage (10 mg/kg); Group 4 (N=6) was given 0.2% cuprizone chow on Day 0, and CNM-Au8 by gavage (10 mg/kg) starting seven days prior to Day 0 when all other groups were started on both cuprizone and CNM-Au8 or vehicle, and continuing through to Day 14. All animals were sacrificed on Day 14 and coronal brain slices were prepared for immunohistochemical staining and quantitation as described in Materials and Methods.

PLP staining and quantitation

7 µm thick serial coronal brain sections between bregma-0.82 mm and bregma-1.82 mm were prepared and analysed. Using previously described methods[3] , paraffin-embedded sections were de-waxed, rehydrated, while housed in a glass container partially filled with 10mM citrate buffer (pH 6.0), and then microwave-heated in a conventional 1.65 KW microwave until the buffer began to boil. Brain sections were then quenched with 0.3% H_2O_2, blocked for 1 hr in PBS containing 3% normal horse serum and 0.1% Triton X-100. Sections were then incubated at 4°C overnight with anti-PLP antibodies (AbD Serotec) at a dilution factor of 1:500. After washing with PBS buffer (pH 7.4), coronal brain sections were further incubated with biotinylated anti-mouse IgG secondary antibody (Vector Laboratories) for 1 hr, followed by exposure to

peroxidase-coupled avidin-biotin complex (ABC Kit, Vector Laboratories) for 30min and developed with diamino-3,3'benzidine reagent according to the manufacturer's instructions (Vector Laboratories). To determine the total cross-sectional area of all brain matter present on each slide, sections were also stained with hematoxylin to label all brain tissue.

Specifically, to quantify the amount of immunopositive PLP in the coronal portion of each mouse brain, coronal sections (i.e., between bregma-0.82mm and 1.82mm) were scanned using a specially adapted Cannon Scanner (output resolution of 2400 dpi). Each pixel intensity in the scan was assigned a numeric intensity value by Photoshop, with "255" corresponding to the least intensity and "1" corresponding to the highest. The data were then exported to Excel. Intensity values were analysed and a quantitative weighted average for intensity (corrected for background by haematoxylin stained area) in each myelin-stained coronal slide was determined.

CNM-Au8 and cuprizone chelation of copper

Materials: cuprizone (Sigma-Aldrich 14960), copper (II) sulphate, 5-hydrate - Macron (4844-04), copper (II) acetate monohydrate (Alfa Aesar A16203), potassium gold(III) chloride (Sigma-Aldrich 334545). 1.0 mM cuprizone solutions were prepared by dissolving solid cuprizone in 50% ethanol. Copper(II) sulphate and copper(II) acetate solutions (10.0 mM) were prepared in MilliQ water. Tris buffered saline (Tris 50.0 mM, NaCl 50.0 mM, pH 7.4) was prepared for spectroscopic experiments.

The copper-cuprizone complex has been previously characterized and is known to have an absorbance peak at 595 nm. An Agilent 8453 spectrophotometer was used to monitor the binding of copper to cuprizone. All samples were blanked with Tris buffer. For saturation curve experiments, final molarity of cuprizone was 8.8×10^{-5} M, and copper concentration was 2.2×10^{-5} M, 4.4×10^{-5} M, and 6.6×10^{-5} M to show the saturation of cuprizone in the cell. Saturation

curves were produced in the presence of CNM-Au8 (26 μg/mL). After adding copper reagents, the samples were incubated for 5 minutes to stabilize the Cu-CPZ complex, and the full spectrum for each sample was recorded.

Rat spinal cord culture and immunohistochemistry

A standard laboratory protocol was followed; in brief, postnatal rat spinal cord cells were dissociated and plated on PLL coated coverslips. Cells were allowed to adhere for at least 24 hours in base medium and then switched to vehicle or CNM-Au8 containing medium (final concentration of 0.01 ug/mL or 10 ug/mL), grown for 72 hours, fixed, and labelled with antibodies as follows: anti-Olig2, anti-MBP, anti-GFAP, anti-CC1.

Supplementary Figures

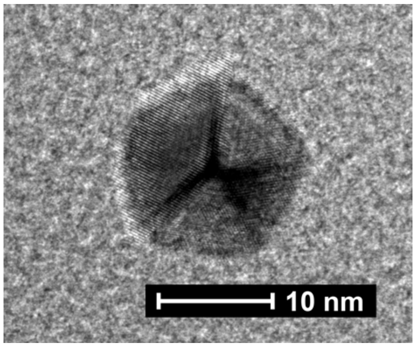

Fig. S1: *TEM image of a representative 13 nm pentagonal bipyramidal gold nanocrystal*

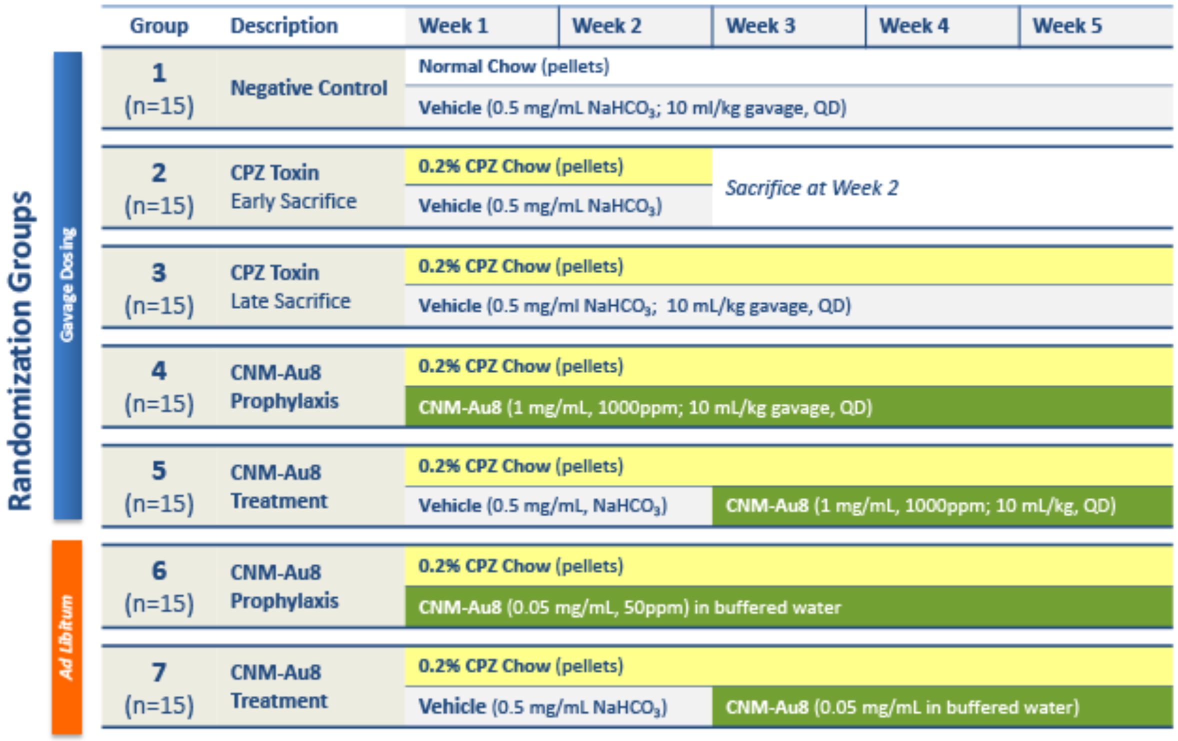

Fig. S2: *Experimental design schematic to demonstrate effect of CNM-Au8 in an in vivo cuprizone model of demyelination. Groups 1-5 were dosed by gavage; Groups 6 and 7 were dosed ad libitum. For Groups 4 and 6, administration of CNM-Au8 began at Week 1, concomitant with cuprizone administration (prophylactic arm); for Groups 5 and 7, administration of CNM-Au8 began at Week 3 (treatment arm).*

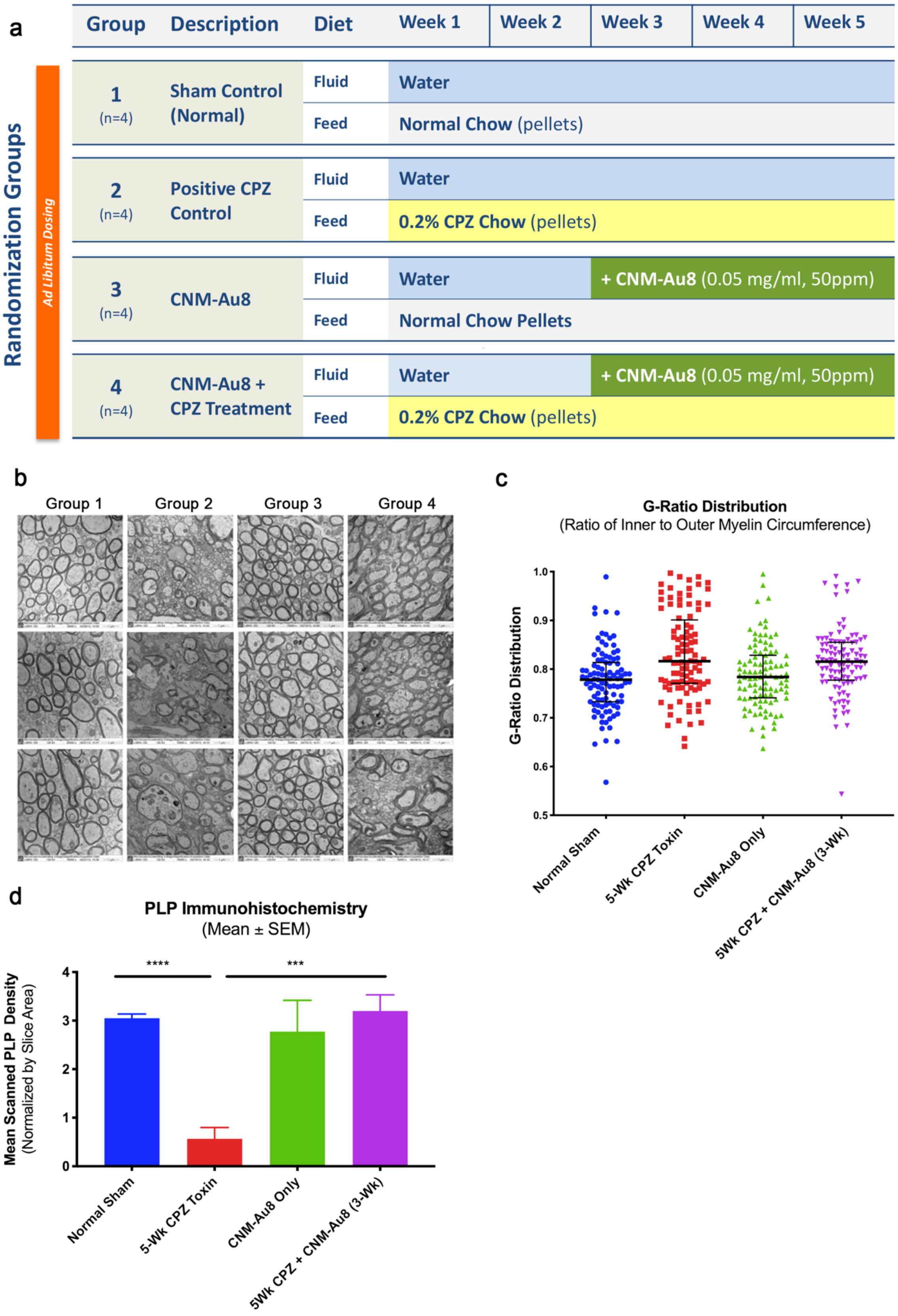
a
Randomization Groups
Ad Libitum Dosing
Group
Description
Diet
Week 1
Week 2
Week 3
Week 4
Week 5
1 (n=4)
Sham Control (Normal)
Fluid
Water
Feed
Normal Chow (pellets)
2 (n=4)
Positive CPZ Control
Fluid
Water
Feed
0.2% CPZ Chow (pellets)
3 (n=4)
CNM-Au8
Fluid
Water
+ CNM-Au8 (0.05 mg/ml, 50ppm)
Feed
Normal Chow Pellets
4 (n=4)
CNM-Au8 + CPZ Treatment
Fluid
Water
+ CNM-Au8 (0.05 mg/ml, 50ppm)
Feed
0.2% CPZ Chow (pellets)
b
Group 1
Group 2
Group 3
Group 4
c
G-Ratio Distribution
(Ratio of Inner to Outer Myelin Circumference)
G-Ratio Distribution
1.0
0.9
0.8
0.7
0.6
0.5
Normal Sham
5-Wk CPZ Toxin
CNM-Au8 Only
5Wk CPZ + CNM-Au8 (3-Wk)
d
PLP Immunohistochemistry
(Mean ± SEM)
Mean Scanned PLP Density
(Normalized by Slice Area)
****
***
4
3
2
1
0
Normal Sham
5-Wk CPZ Toxin
CNM-Au8 Only
5Wk CPZ + CNM-Au8 (3-Wk)

Fig. S3: *Evidence of remyelination by CNM-Au8 in a pilot in vivo cuprizone mouse model.* (***a***) *Experimental design schematic.* (***b***) *Three representative TEM images from the corpus callosum of mice from each group (16,000x magnification).* (***c***) *G-ratio distribution scatter plots of 100 randomly selected axons from images of corpus callosum sections of one animal from each group, assessed in blinded fashion. Bars show the median with interquartile ranges. Smaller g-ratios indicate thicker myelin sheaths.* (***d***)*, Quantitation of anti-PLP staining of coronal sections of whole brain of one animal from each group, normalized by haematoxylin-stained area. *** p value < 0.001 using two-tailed Student's t-test comparison to 5-wk cuprizone toxin (Group 2).*

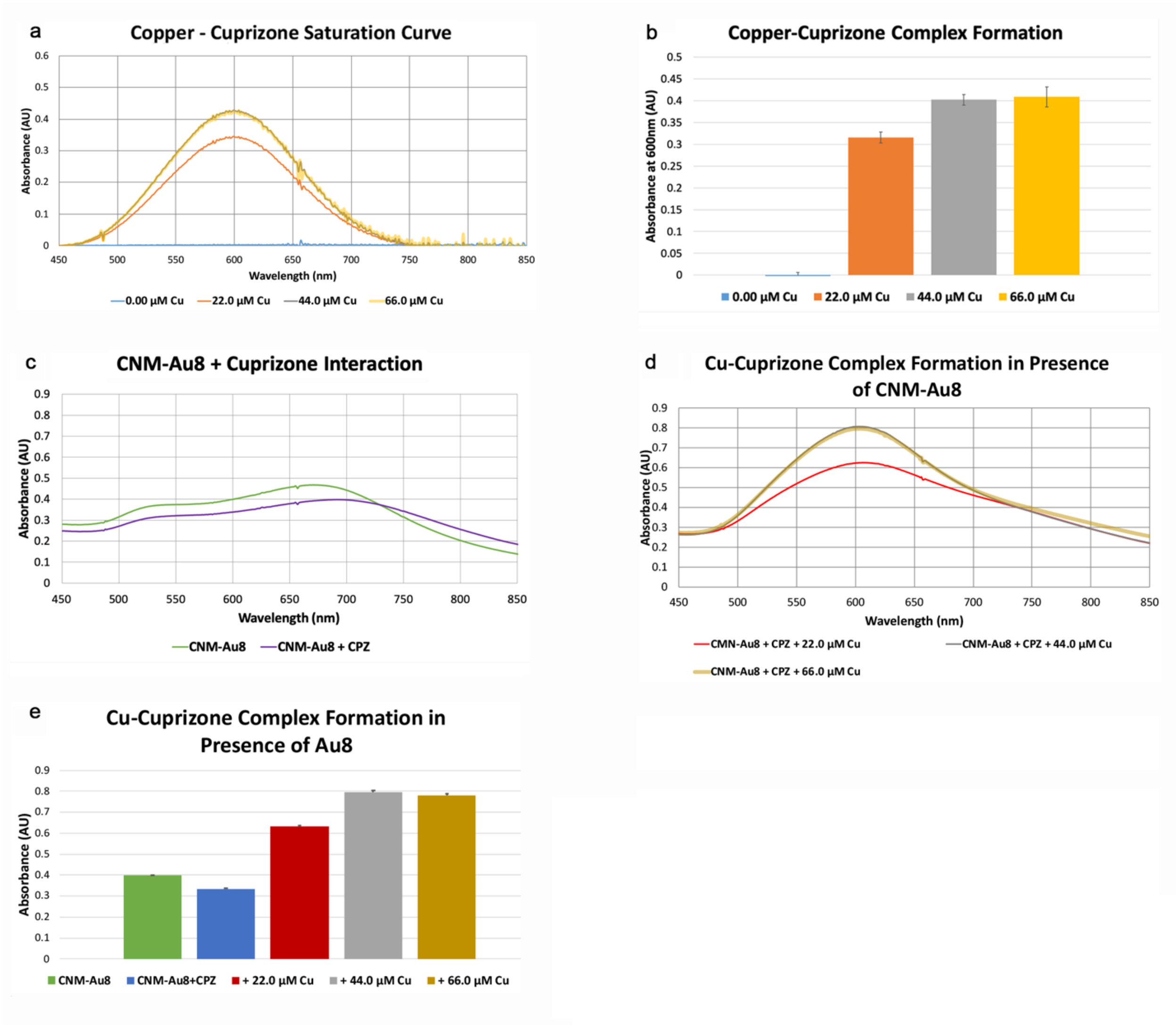

*Fig. S4: CNM-Au8 did not inhibit cuprizone from chelating copper. (**a**) Copper-cuprizone complexes had a broad absorbance peak at 600 nm, observed upon the addition of varying amounts of Cu(II) sulphate to 88.0 µM cuprizone. Cuprizone became saturated on the addition of 44.0 µM Cu (grey), the curve for which is superimposed by the curve of 66.0 µM Cu (yellow). (**b**) Quantitation of the average absorbance of the Cu-cuprizone complex at 600 nm (shown in A) for triplicate samples. (**c**) Minimal changes in the UV-Vis absorbance profile of 26 µg/mL CNM-Au8 (~2 µM) in the presence (purple) or absence (green) of 88.0 µM cuprizone indicated a lack of binding interaction between CNM-Au8 and cuprizone. The slight spectral shift may indicate possible minor surface interactions. (**d, e**) CNM-Au8 did not interfere with Cu-cuprizone binding. Saturation of 88.0 µM cuprizone occurred with the addition of 44.0 µM copper (grey)*

*in the presence of 26 μg/mL CNM-Au8. (**e**), Quantitation of the average absorbance of the Cu-cuprizone complex in the presence of 26 μg/mL CNM-Au8 at 600 nm (shown in **d**) for triplicate samples. Saturation of cuprizone occurred with the addition of 44.0 uM copper (grey).*

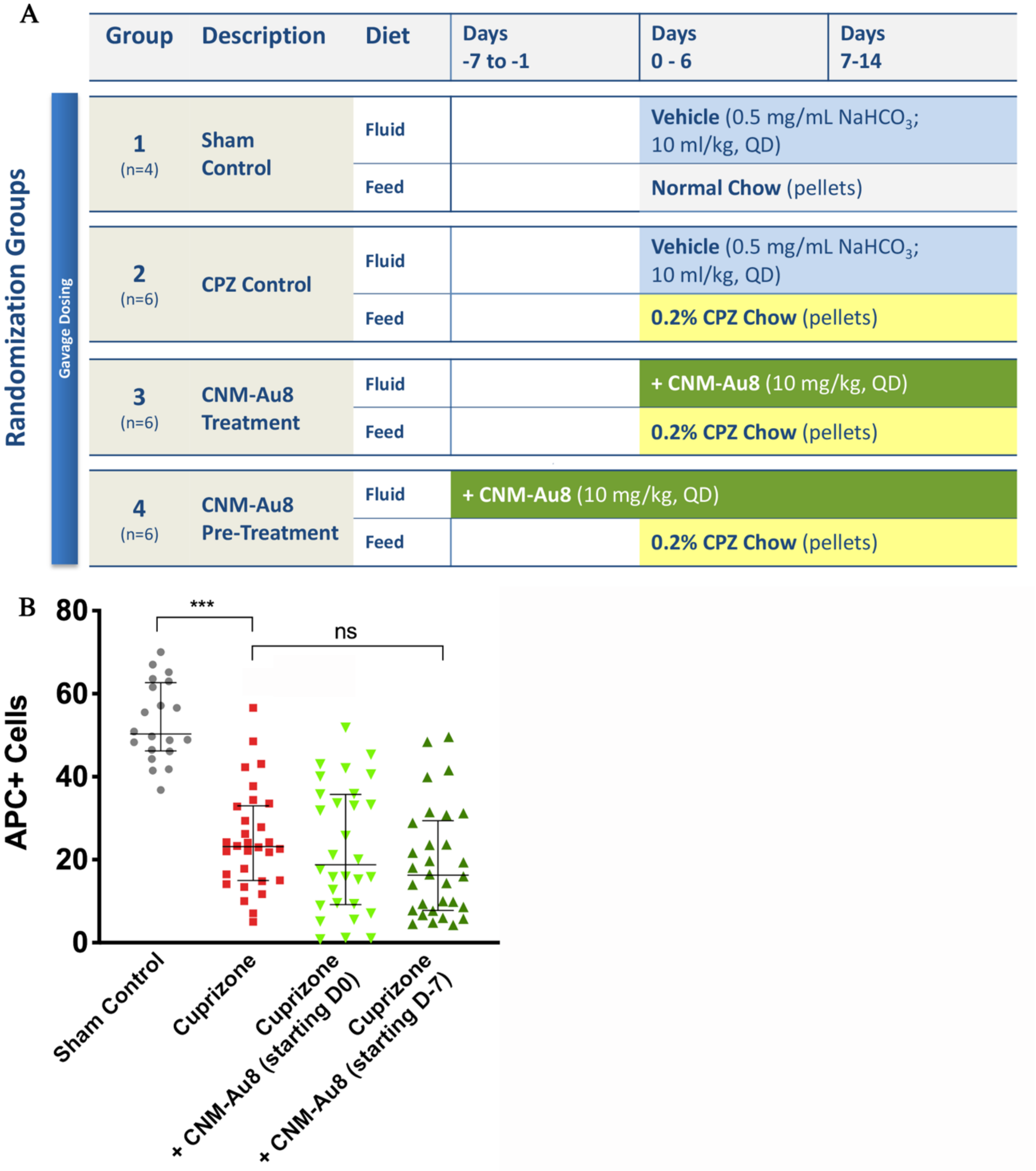

Fig. S5. *Loss of Apc+ OLs was unaffected by CNM-Au8 treatment within the first two weeks of cuprizone treatment. (**a**) Study schematic. (**b**) Quantitation of the number of APC positive cells by immunohistochemical staining of coronal sections from each treatment group. Error bars show median and interquartile range.*

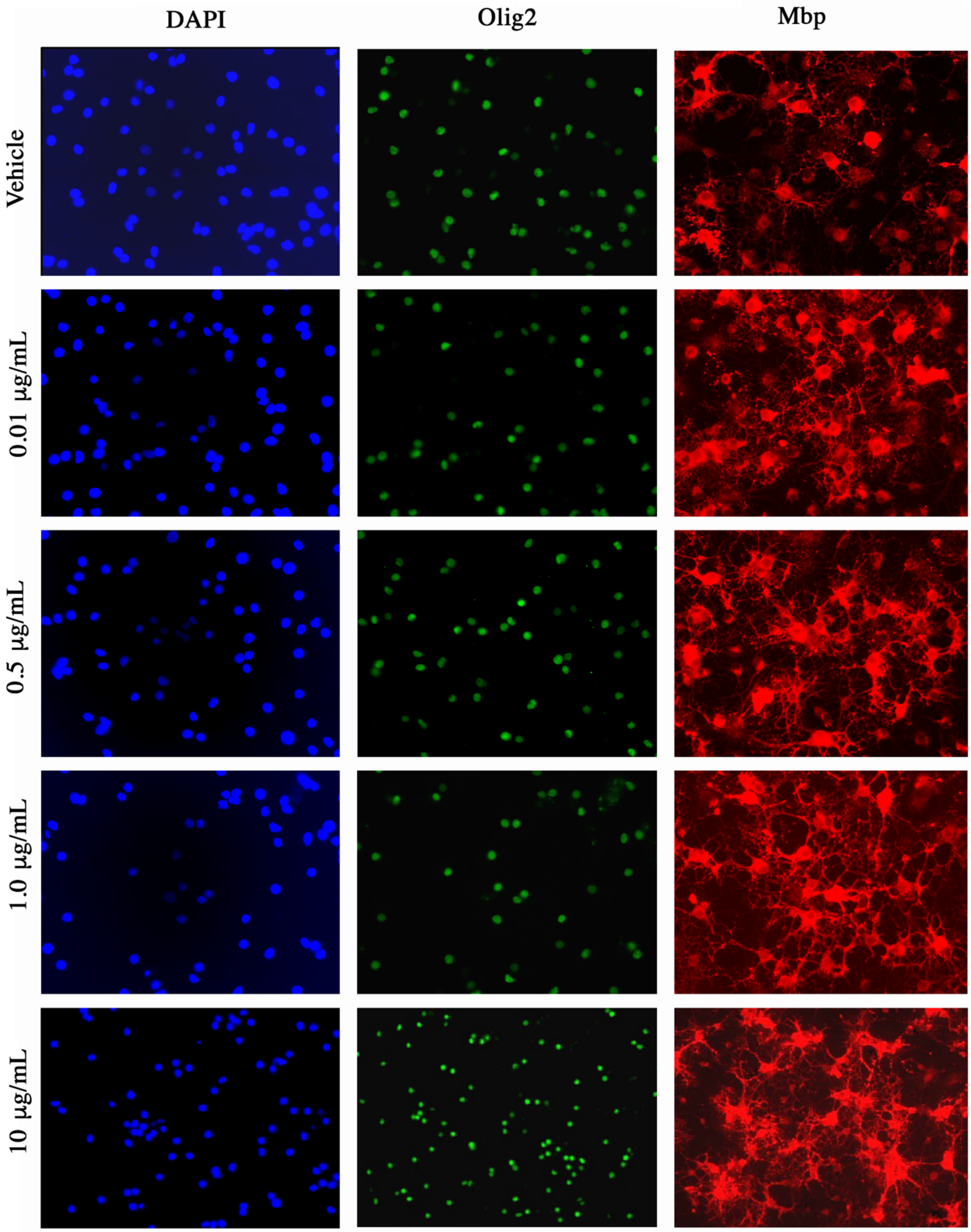

Fig. S6. *Differentiation of OLs from primary rodent spinal cord cultures by CNM-Au8. Postnatal spinal cord cells were cultured for 72 hours in the presence of vehicle or doses of CNM-Au8 (0.01 µg/mL, 0.5*

μg/mL, 1.0 μg/mL. and 10 μg/mL). Cells were then fixed and stained with DAPI, anti-Olig2 antibodies, and anti-Mbp antibodies. Increasing levels of Mbp expression as well as an increase in OL 'footprint' can be observed in association with higher levels of CNM-Au8 treatment.

Table S1. CNM-Au8 nanocrystal characterisation

Characteristic	Determination	Analytical Procedure
**Appearance**	Dark Red/Purple liquid	UV-Vis spectra
**Distribution of three-dimensional shapes**	25% pentagonal bipyramid, 23% tetrahedron, 6% octahedron, 23% hexagonal, 23% other	Transmission electron microscopy
**Nanocrystal Size**	Hydrodynamic radius = 10–30 nm	Dynamic Light Scattering
**Identity**	λ_{PEAK} between 515 - 540 nm	Optical absorbance via UV-Vis spectrophotometry
**UV-Vis Ratio**	$Abs_{450}/Abs_{650} \geq 1.6$	Optical absorbance via UV-Vis spectrophotometry
**Zeta Potential**	Zeta potential ≤ -30 mV	Electrophoretic Mobility
**pH**	7.5–10.5	pH

Table S2. *Functional improvements in each open field parameter toward Sham controls by CNM-Au8 treated groups compared to vehicle controls. RSE, relative standard error.* [a] *p = 0.12.* * *p=0.04, two-tailed t-test, corrected for multiple comparisons.*

	**CNM-Au8 plus Cuprizone, % of sham (RSE)**	**Vehicle plus Cuprizone, % of sham (RSE)**	**Percent Relative Improvement of CNM-Au8 Treatment vs. Vehicle (Toward Sham)**
**Path length**	93.17 (-0.03)	89.02 (-0.03)	62%
**Central Path Length**	72.65 (0.09)	51.19 (-0.10)	56% [a]
**Vertical Rearing**	89.03 (-0.03)	80.54 (-0.04)	56% [a]
**Central Vertical Rearing**	67.45 (-0.13)	24.41 (-0.15)	43% *
**Jump Count**	87.50 (-0.05)	79.23 (-0.05)	60%
**Jump Time**	94.25 (-0.04)	87.87 (-0.05)	47%

Table S3. *RNASeq DE genes identified overrepresenting the pathways shown in Fig. 7c.*

GO.ID	Term	Annot	Signif	Expected	classicFisher	elim	sigGenes	geneSet
**GO:0016021**	integral component of membrane	3079	111	68.26	1.1e-08	1.1e-08	1110007C09Rik, 2310044H10Rik, 3110056O03Rik, Aatk, Abca2, Abcd1,Adra1d, Alg3, Aplp1, Atf6b, Atg9a, Atp13a2, B930041F14Rik, Bcam, C1qtnf1, C3, Car14, Cdh22, Chpf, Chst12, Cldn14, Cldn19, Cmtm5, Cyp46a1, Ddr1, Efnb3, Elovl1, Fam38a, Fam73b, Fkbp8, Gal3st1, Gdpd5, Gjb1, Gjc2, Gm16517, Gpaa1, Gpr137, Grina, H2-D1, Hcn2, Ifitm2, Igsf8, Il12rb1, Ints1, Itgb4, Jph3, Kcnab2, Kcnn1, Kirrel3, Lemd2, Lrfn3, Lrfn4, Mag, Mfsd3, Mospd3, Mpv17l2, Ncln, Ngfr, Ninj1, Olfm2, Osbp2, Paqr4, Pcdh1, Pllp, Ppap2c, Prr7, Ptchd2, Ptges2, Ptprn, Rabac1, Relt, Rtn2, S1pr5, Scamp4, Scn1b, Sema6c, Sez6, Sidt2, Slc25a10, Slc25a29, Slc25a39, Slc27a4, Slc29a4, Slc35b2, Slc35c2, Slc35e4, Slc39a3, Slc45a3, Spns1, Stard3, Steap3, Svop, Syngr2, Tmc6, Tmem115, Tmem132a, Tmem134, Tmem143, Tmem151a, Tmem160, Tmem180, Tmem198, Tmem205, Tmem63a,	UP

							Tmem98, Tspan9, Tyro3, Unc5b, Wscd1, Yif1b, Zdhhc12	
**GO:0042552**	myelination	115	12	2.51	7.8e-06	0.00218	Gal3st1, Gpc1, Mag, Mbp, Ndrg1, Ngfr, Nkx6-2, Pllp, Rxrb, Sh3tc2, Sox10, Trf	UP
**GO:0000038**	very long-chain fatty acid metabolic processes	24	3	0.52	0.01485	0.01485	Abcd1, Elovl1, Slc27a4	UP
**GO:0005886**	plasma membrane	2981	89	66.09	0.00107	0.00107	2310035K24Rik, 3110056O03Rik, Adap1, Adra1d, Alg3, Ankrd13b, Apbb1, Aplp1, Arap1, Arc, Atp13a2, Bcam, Bcar1, C1qtnf1, Cdc42ep1, Cdc42ep2, Cdh22, Cldn14, Cldn19, Dcxr, Ddr1, Diras1, Dnm1, Eef2, Efnb3, Ehd1, Eno2, Fam38a, Fam73b, Fasn, Fscn1, Gjb1, Gjc2, Gnb2, Gng7, Gpaa1, Gpc1, Gsn, H2-D1, Hcn2, Hdac5, Hmox1, Ifitm2, Igsf8, Il12rb1, Itgb4, Jph3, Kcnab2, Kcnn1, Kirrel3, Ldlrap1, Lrfn3, Lzts2, Maf1, Mag, Mapk3, Mbp, Mfsd3, Mpp2, Ndrg1, Ngfr, Olfm2, Pcdh1, Phlda3, Plch2, Ppap2c,	UP

							Prosapip1, Prr7, Ptprn, Rabac1, Relt, Rhog, Rtn2, Rusc1, S1pr5, Scn1b, Sema6c, Sez6, Sh3tc2, Sidt2, Slc27a4, Slc29a4, Slc9a3r2, Steap3, Tmem198, Trf, Tspan9, Tyro3, Unc5b	
**GO:0006739**	NADP metabolic process	35	3	0.76	0.04032	0.04032	Dcxr, Kcnab2, Pgls	UP
**GO:0031982**	vesicle	2987	90	66.22	0.00073	0.13135	2310035K24Rik, 3110056O03Rik, Aatk, Abca2, Abhd8, Adamtsl4, Ankrd13b, Arap1, Arc, Arhgap23, Arl2, Atg9a, Atp13a2, Bag6, Bcam, C3, Chga, Ctsd, Dcxr, Ddr1, Dnm1, Eef2, Ehd1, Eno2, Fasn, Fbxo2, Fscn1, Ftl1, Gaa, Gamt, Gnb2, Gng7, Gpc1, **Gpx4**, Gsn, H2afj, H2-D1, Hist1h4h, Igsf8, Islr, Itgb4, Ldlrap1, Lzts2, Map1lc3a, Mapk3, Naglu, Ndrg1, Ngfr, Padi2, Pcsk1n, Pgls, Phlda3, Plbd2, Pllp, Plod1, Plod3, Ppfia3, Ppt2, Ptchd2, Ptprn, Rab1b, Rabac1, Relt, Rhog, Rusc1, Rusc2, Scamp4, Serpinf1, Sh3bgrl3, Sh3tc2, Slc9a3r2, Snx15, Spns1, Stard3, Steap3, Svop, Syn1, Syngr2, Tbc1d17, Tmc6, Tmem132a, Tmem198, Tmem205, Tmem63a, Tppp3, Trf, Tubb3, Urm1, Wdr81, Yif1b	UP

**GO:0044304**	main axon	68	7	1.51	0.00075	0.00075	Apbb1, Dnm1, Gjc2, Kcnab2, Mag, Mbp, Scn1b	UP
**GO:0030054**	cell junction	1034	37	22.92	0.00255	0.00255	Arc, Arl2, Bcar1, Cdc42ep1, Cldn14, Cldn19, Fscn1, Git1, Gjb1, Gjc2, Gnb2, Gsn, Itgb4, Kcnab2, Lrfn3, Mapk3, Mpp2, Mtap1s, Ndrg1, Ngfr, Olfm2, Pcdh1, Prosapip1, Prr7, Ptprn, Rabac1, Rhog, Rusc1, Scn1b, Sez6, Slc9a3r2, Sorbs3, Stard10, Svop, Syngr2, Tspan9, Zyx	UP
**GO:0035639**	purine ribonucleoside triphosphate binding	1459	51	32.31	0.00062	0.03579	Abce1, Acsl4, Actr2, Akt3, Arl4a, Atad1, Atp13a3, Bmpr1a, Cct4, Cdk14, Chuk, Clk1, Cmpk1, Dars, Dclk1, Ddx3x, Dhx36, Dnaja1, Dnm1l, Eif4a2, Eif5, Gnai1, Gnai3, Hsp90aa1, Hsp90b1, Hspa4, Hspd1, Jak2, Map4k5, Npm1, Pak3, Pank3, Psmc6, Rab1, Rab11a, Rab18, Rab8b, Rap1a, Rap1b, Rap2c, Rragd, Sar1b, Sept2, Sept7, Skiv2l2, Smarca5, Sucla2, Top2a, Top2b, Uba3, Yme1l1	DOWN
**GO:0017076**	purine nucleotide binding	1503	51	33.29	0.00121	0.05317	Abce1, Acsl4, Actr2, Akt3, Arl4a, Atad1, Atp13a3, Bmpr1a, Cct4, Cdk14, Chuk, Clk1, Cmpk1, Dars, Dclk1, Ddx3x, Dhx36, Dnaja1, Dnm1l, Eif4a2, Eif5, Gnai1, Gnai3, Hsp90aa1, Hsp90b1, Hspa4, Hspd1, Jak2, Map4k5, Npm1, Pak3, Pank3, Psmc6, Rab1, Rab11a, Rab18, Rab8b, Rap1a, Rap1b, Rap2c, Rragd, Sar1b,	DOWN

							Sept2, Sept7, Skiv2l2, Smarca5, Sucla2, Top2a, Top2b, Uba3, Yme1l1	
**GO:0019904**	protein domain specific binding	683	35	15.13	3.1e-06	3.1e-06	Adam9, Cacybp, Cadm1, Calm2, Capn7, Chmp1b, Crk, Ctnnb1, Cul3, Ddc, Dnaja1, Dnm1l, Dock4, Fmr1, Gnai3, Hdac2, Hif1a, Hsp90aa1, Jak2, Lin7c, Npm1, Pak3, Rab8b, Rabep1, Sh3bgrl, Skil, Skp1a, Srsf1, Srsf7, Stag1, Strn3, Tceal8, Tra2b, Usp47, Wbp5	DOWN
**GO:0031461**	cullin-RING ubiquitin ligase complex	160	13	3.61	6.8e-05	6.8e-05	Anapc4, Btbd1, Cacybp, Cdc27, Crbn, Cul2, Cul3, Cul4b, Klhl13, Klhl7, Klhl9, Skp1a, Usp47	DOWN
**GO:0005654**	nucleoplasm	2210	75	49.83	0.00012	0.00012	Acbd5, Appl1, Arl4a, Bmi1, Bnip3l, Cacybp, Capn7, Cct4, Cdc27, Cfdp1, Cggbp1, **Chuk**, **Creb1**, Cse1l, Cul2, Cul4b, Dld, Eif3e, Fabp7, Fmr1, Fyttd1, Hdac2, Hif1a, Hnrnph1, Hpgd, Htatsf1, Ipo7, Jak2, Klhl7, Kpna3, Kpna4, Luc7l3, Mocs2, Morf4l2, **Nampt,** Nasp, Ncl, Npm1, Orc2, Pcnp, Phf6, Pnrc2, Polr3k, Psip1, Ptges3, Rab8b, Rbm39, Rlim, Rnf13, Rragd, Sdcbp, Skil, Smarca5, Srsf1, Srsf10, Srsf3, Srsf7, Stag1, Strn3, Stxbp3a, Supt16h, Syap1, Tardbp, Tceb1, Top2a, Top2b, Topbp1, Tra2b, Trim37, Trove2,	DOWN

							Yme1l1, Ythdc1, Zbtb33, Zfp260, Zzz3	
